# Textured ceramic membranes for desilting and deoiling of produced water in the Permian Basin

**DOI:** 10.1016/j.isci.2022.105063

**Published:** 2022-09-05

**Authors:** Natalia Rivera-Gonzalez, Aayushi Bajpayee, Jakob Nielsen, Umme Zakira, Wasif Zaheer, Joseph Handy, Tiffany Sill, Bjorn Birgisson, Mukul Bhatia, Sarbajit Banerjee

**Affiliations:** 1 Department of Chemistry, Texas A&M University, College Station, TX 77843-3255, USA; 2 Department of Materials Science and Engineering, Texas A&M University, College Station, TX 77843-3255, USA; 3 Zachry Department of Civil & Environmental Engineering, Texas A&M University, College Station, TX 77843-3136, USA; 4 Department of Geology and Geophysics, Texas A&M University, College Station, TX 77843-3115, USA; 5 Berg-Hughes Center for Petroleum & Sedimentary Systems, Texas A& M University, College Station, TX 77843-3115, USA

**Keywords:** Catalysis, membranes, engineering

## Abstract

Oil production in the Permian Basin gives rise to large volumes of produced water contaminated by silt, emulsified oil, and additives used for enhanced oil recovery. There is intense interest in the design of membrane modules as sustainable alternatives for produced water treatment to enable the reuse of produced water for agricultural applications, injection into aquifers, and redeployment in oil recovery. Here, we report a hierarchically textured cement-based membrane exhibiting orthogonal wettability, specifically, superhydrophilic and underwater superoleophobic characteristics. The *in situ* formation of ettringite needles accompanied by embedding of glass spheres imbues multiscale texturation to stainless-steel mesh membranes, enabling the separation of silt and oil from produced water at high flux rates (1600 L h^−1^۰m^−2^, at ca. 2.7 bar). Oil concentration is reduced as low as 1 ppb with an overall separation efficiency of 99.7% in single-pass filtration. The membranes show outstanding mechanical resilience and retention of performance across multiple cycles.

## Highlights

• Multiscale 3D texturation of a metal mesh membrane engenders orthogonal wettability
• High separation efficiency of ca. 99% was achieved for produced water
• Oil concentration reduced to ≤1 ppb in a single pass at a high flux rate
• Desiliting and deoiling demonstrated for real produced water streams

## Graphical abstract

**Figure:**
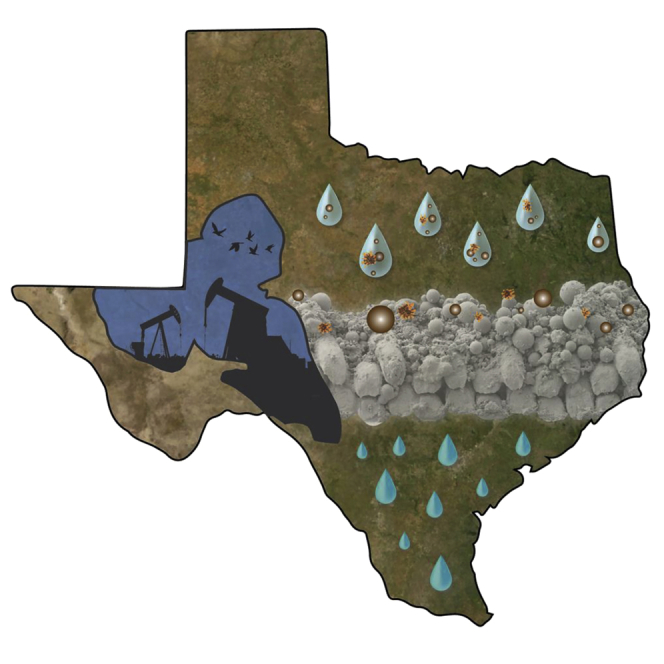

## Introduction

The Permian Basin, which extends across a vast swathe of West Texas and Southwest New Mexico, has emerged as a leading oil and gas producing region with daily production approaching 4.5 million barrels of oil (EIA, 2021; Wang, 2020; Scanlon et al., 2021). This represents nearly half of domestic production in the United States (Wang, 2021; Scanlon et al., 2021). A major emerging concern in the Permian Basin stems from the large volumes of produced water (PW) generated as a waste-stream in extraction processes (Dickhout et al., 2017; Igunnu and Chen, 2012). This complex oil recovery byproduct contains a diverse range of contaminants, including high concentrations of total dissolved solids (TDS), emulsified oil and grease, silt, endogenous surfactants, and additives from enhanced oil recovery operations. The specific composition of PW is highly variable and depends on the geologic age, depth, location, and specific geochemistry of the formation; the chemical composition of hydrocarbons extracted from the reservoir; the extraction method; and additives used for enhanced oil recovery (Neff et al., 2011; Rodriguez et al., 2020).

Scanlon and co-workers estimate that between 2005 and 2015, PW generated from conventional and unconventional reservoirs in the Permian Basin amounted to ca. 40 × 10^9^ bbl and 4 × 10^9^ bbl, respectively (Scanlon et al., 2017). Production of PW from unconventional oil and gas reservoirs has steadily increased in this semi-arid region, giving rise to water scarcity concerns. Although conventional reservoirs produce ca. 13 times more water than oil as compared to unconventional reservoirs, their high permeability enables the PW to be re-injected and used for enhanced oil recovery (Scanlon et al., 2017). In contrast, PW from unconventional reservoirs, while requiring relatively less water during extraction (a PW-to-oil ratio of ca. 3 is typical), represents a major challenge for disposal given the low permeability of shale reservoirs. Current disposal methods involve the injection of PW in non-oil-producing constructs such as salt-water disposal wells. PW disposal represents an existential challenge for the energy industry and is directly linked to water scarcity, induced seismicity, contamination of freshwater resources, and groundwater methane contamination (Scanlon et al., 2020a, 2020b). As such, there is intense interest in the design of technologies for cleaning PW to enable its reuse for oil recovery as well as in agricultural, drinking water, and hydrological applications (Neff et al., 2011; Jiménez et al., 2018). Conventional PW management systems entail multi-stage treatment trains. Such treatment trains include primary/secondary treatments such as API separation, coagulation, and air/gas flotation; “polishing” approaches such as biological treatment, media filters, and membrane technology; and tertiary treatments such as adsorption and microfiltration/ultrafiltration (Dickhout et al., 2017; Jiménez et al., 2018; Igunnu and Chen, 2012). These flow treatments can help reduce the oil content and salinity of the PW. However, treatment trains have not been widely adopted as a result of their large footprints, propensity for producing significant volumes of sludge, and prohibitive capital and maintenance costs (Mercelat et al., 2021; Fakhru’l-Razi et al., 2009). Notably, there is considerable variation of PW across different regions of the Permian Basin (Cooper et al., 2021), which renders the design of universal treatment trains rather challenging. Innovative water treatment solutions are urgently required to address the challenges of PW treatment and to ensure a supply of fresh water for agricultural and industrial purposes in the semi-arid Permian, which is home to several growing cities.

Membrane-based technologies can potentially provide a viable alternative for PW management at a relatively lower cost and higher throughput. These technologies hold promise for improved water quality, compact design, facile operation, and low energy consumption (Cai et al., 2021; Babayev et al., 2019). An effective membrane module can greatly enhance the efficiency of PW treatment trains. In designing a membrane for PW treatment, it is imperative to consider (i) the characteristics of the emulsion sought to be separated; (ii) interfacial interactions at the solid/liquid boundary; and (iii) process conditions and reactor design, with a view toward optimizing flow properties and system performance (Douglas et al., 2022). When building membrane architectures to destabilize complex emulsions and engender separations, the wettability of the surface is of paramount importance. The observed wettability reflects a complex interplay of surface chemistry and nano/micron-scale topological features (Padaki et al., 2015; Dickhout et al., 2017). Accessing superwetting characteristics to engender the separation of oil and silt particles from PW requires textured surfaces with hierarchical macro-/nanotexturation. A wide variety of materials have been evaluated as the active elements of membrane-based technologies; however, they commonly suffer from fouling, low flux rates, and poor chemical/thermal stability (Olajire, 2020; Zolghadr et al., 2021). As an example, we have previously reported membrane architectures based on ZnO tetrapods for separating viscous oil emulsions based on principles of orthogonal wettability (O’Loughlin et al., 2018). However, such membranes are somewhat constrained in terms of achievable flow rates. Recent work has examined a variety of nanostructured substrates (e.g., hollow fibers with TiO_2_, graphene oxide, date seed biomass) for deoiling and PW treatment and achieved promising separation (Zou et al., 2022; Alammar et al., 2020, 2022). Ceramic membranes have attracted particular attention owing to their combination of mechanical, chemical, and thermal stability (Padaki et al., 2015; Zoubeik et al., 2019; Zolghadr et al., 2021; Hussain et al., 2020), as well as prospects for being able to withstand high pressures while yielding reasonable flow rates (Bolto et al., 2020). Ceramic-based membrane systems offer a distinctive combination of low cost, scalability, high flux, enhanced performance, low contamination, and high thermal stability. The major challenge to the implementation of such systems is the hydration time, which nevertheless can be addressed through off-site manufacturing and selection of fast-hydration cement chemistries. Additionally, as with any cement-based materials, such membranes are unsuitable for application in highly acidic media.

In this work, we report the design of membrane architectures based on calcium sulphoaluminate (CSA) cement and glass spheres (GS) to clean PW from the Permian Basin. Specifically, we examine the multivariate dependence of CSA-based membranes considering membrane composition, thickness, and pore dimensions. The membranes exhibit a combination of underwater superoleophobic and superhydrophilic character, enabling the effective separation of silt and oil. The oil concentration in filtered PW samples is reduced to several ppb in a single pass while maintaining a high flux rate approaching 1600 L h^−1^۰m^−2^. CSA is a low-cost structural material that has a fast-setting time, high early strength, and substantially reduced carbon footprint as compared to ordinary Portland cement (Zhou et al., 2006; Hargis et al., 2014). These characteristics make it viable as a membrane component that is manufacturable at scale.

## Results and discussion

### Design and characterization of calcium sulphoaluminate/and glass spheres membrane architectures for desilting and deoiling

**Figure 1:**
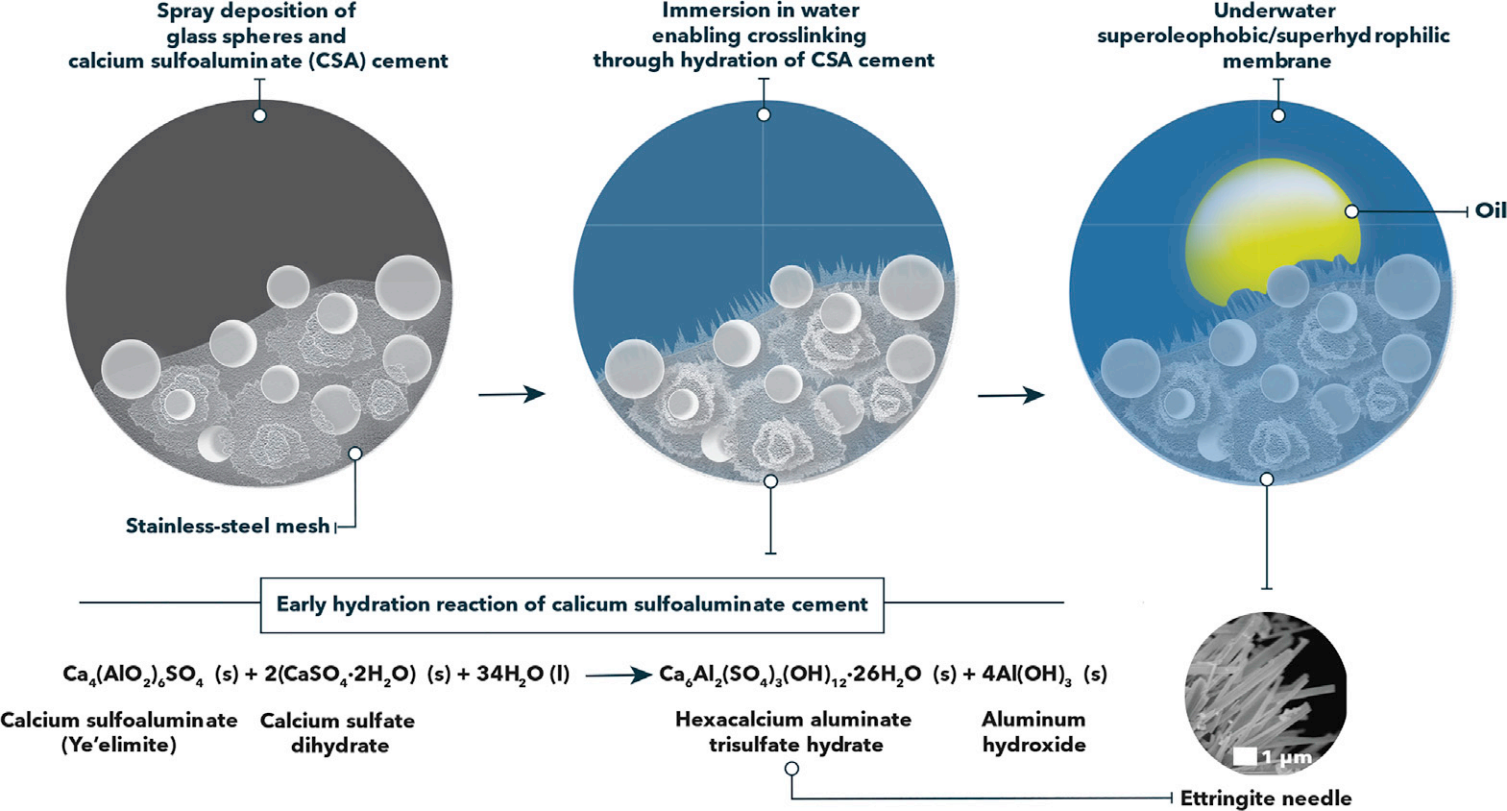
Schematic depiction of the synthetic approach for the fabrication of CSA-based membranes CSA cement and glass sphere particles are spray-coated onto stainless steel meshes with micrometer-sized pores (5 μm), followed by the hydration of CSA cement through the immersion of the membrane architecture in water to initiate and enhance the extent of crosslinking at the surface of the membrane. This process yields an underwater superoleophobic/superhydrophilic surface. The hydration process of CSA cement delivers hexacalcium aluminate trisulfate hydrate or “ettringite” as the primary phase. After 24 h, well-defined needles of “ettringite” are formed.

**Figure 2:**
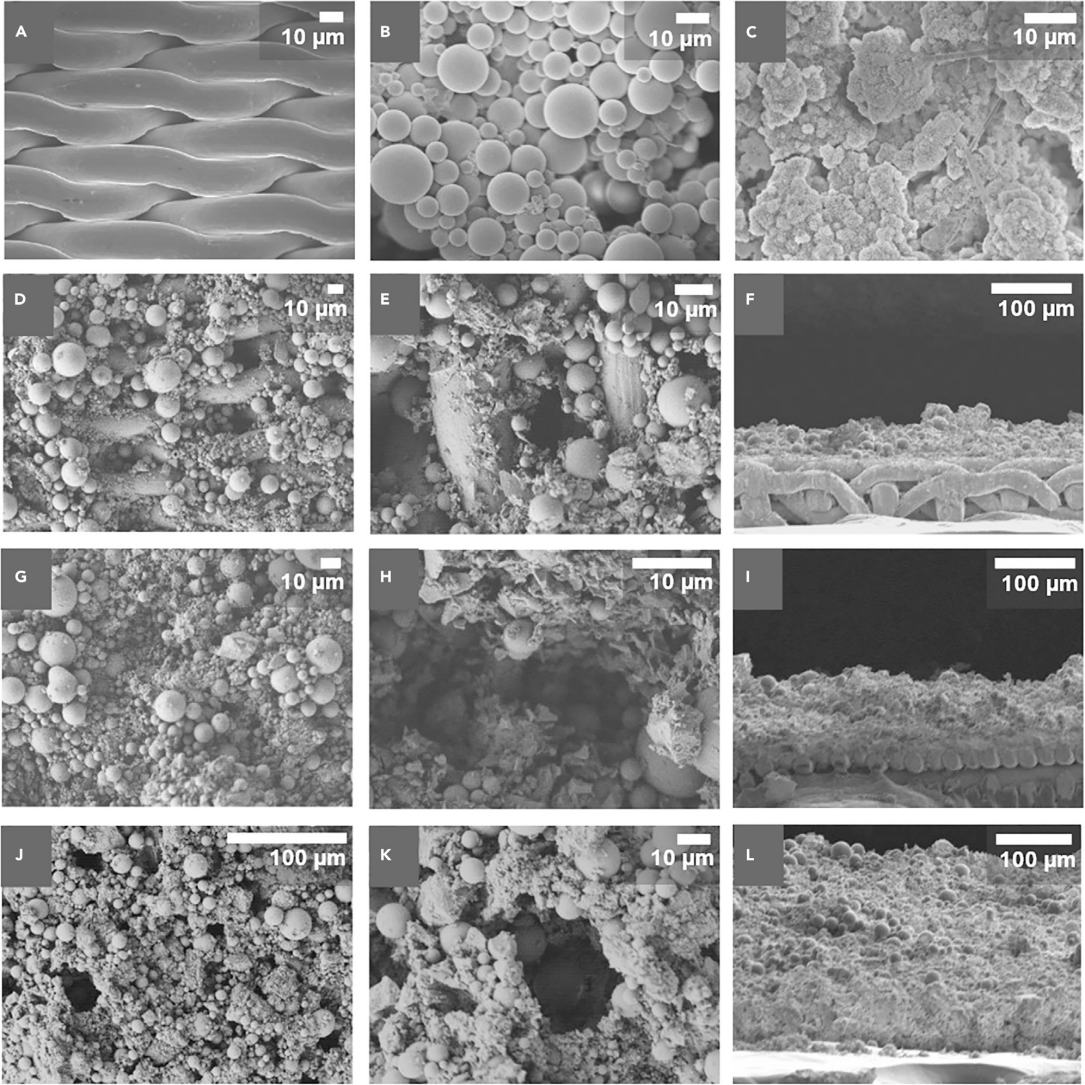
Representative SEM images depicting CSA/glass-bead-coated membranes with different proportions of CSA to glass sphere and varying surfaces thicknesses (A) Stainless-steel mesh with 5 μm pore edge; (B) SiO_2_ particles; (C) microstructure of hydrated CSA without the addition of glass beads; (D-E) CSA/GS membranes with a 1:1 (w/w) CSA/GS loading totaling 5 mg cm^−2^; (F) cross-sectional view of membrane with 5 mg cm^−2^ of 1:1 (w/w) CSA/GS loading at a thickness of 55 ± 10 μm; (G and H) CSA/GS membrane with a 2:1 (w/w) ratio totaling a loading of 10 mg cm^−2^; (I) cross-sectional view of membrane with 10 mg cm^−2^ of 2:1 (w/w) CSA/GS loading at a thickness of 102 ± 9 μm; (J and K) CSA/GS membrane with a 4:1 (w/w) loading totaling 20 mg cm^−2^; (L) cross-sectional view of a membrane with 20 mg cm^−2^ of 4:1 (w/w) CSA/GS loading at a thickness of 207 ± 35 μm. Scale bars, 10 μm and 100 μm.

In this article, we have sought to achieve the de-emulsification of PW by leveraging principles of orthogonal wettability. We have demonstrated in previous studies that membrane-based structures containing ZnO tetrapods yield hierarchically textured surfaces that allow for the separation of viscous oil and water (O'Loughlin et al., 2017; Bajpayee et al., 2019). In considering the separation of recalcitrant emulsions from the Permian Basin, the membrane structure should enable the selective permeation of the aqueous phase while rejecting oil droplets by dint of its surface energy. It should furthermore retain silt particles greater than the pore diameter. Given that PW treatment membranes need to operate under complete liquid immersion, we have sought to design superhydrophilic and underwater superoleophobic architectures (Chen et al., 2016). Figure 1 illustrates the workflow for preparing hierarchically textured CSA-based membranes. CSA and glass spheres are sprayed onto stainless-steel mesh substrates with pore dimensions of 5 × 5 μm; the mesh imbues micron-scale texturation and serves as a continuous support for the otherwise brittle cement thin film. Figure 2 illustrates the porous network that is stabilized with nanotexturation derived from arrays of ettringite needles formed on hydration (Figure 1). During the hydration of CSA cement, hexacalcium aluminate trisulfate hydrate (“ettringite”) and aluminum hydroxide are formed as the primary hydration products (*vide infra*). The growth of a crystalline network of needle-like crystals is observed almost immediately on exposing CSA cement to water. The microporosity of the surface is defined by the spacing between the ettringite needles and hydrated CSA matrix. The morphology and size distribution of the ettringite needles are governed by the (i) activity of water inside the cement pore; (ii) degree of hydration; and (iii) the local environment of hydration products. The hydration reaction that yields ettringite is mostly completed within 24 h. The thickness, as well as the length of the needles, increases with time; hydration is entirely completed within 7 days (Baur et al., 2004).

The workflow sketched in Figure 1 can be readily tuned to control pore dimensions and thickness, enabling the systematic evaluation of trade-offs between flux rate and selectivity while ensuring mechanical resilience. Figure 2 shows representative scanning electron microscopy (SEM) images of substrates illustrating the porous microstructures obtained for varying proportions of CSA to glass spheres at different coating thicknesses. The glass spheres embedded in the CSA matrix range in size from 9 to 13 μm and are spray-coated onto stainless steel meshes, which have pore dimensions of 5 × 5 μm, as illustrated in Figures 2A-2C. The weight proportions of CSA and glass beads have been altered to systematically vary the porosity of the coating microstructure, and thus the effective tortuosity of the membranes (Figures 2D, 2G, and 2J); furthermore, the total loading of CSA and glass beads has been varied to control the coating thickness, as illustrated by the cross-sectional SEM images in Figures 2F, 2I, and 2L. At a CSA:glass sphere weight ratio of 4:1 and a total loading of 20 mg cm^−2^, the dimensions of the porous network are highly constricted and the 3D pore structure allows for considerable interfacial interactions. Substantial 3D texturation, as well as tortuosity, is evident in Figures 2E, 2H, and 2K. With an increase in total loading from 5 to 20 mg cm^−2^, the coating thickness increases from 55 ± 10 μm to 207 ± 35 μm.

American Society for Testing Material (ASTM) D2197 scrape adhesion testing has been performed for the CSA/GS coatings and yield a rating of 100 g on hydration for 24 h and 150-200 g on hydration for 28 days (Figure S1 in the supplemental information).

**Figure 3:**
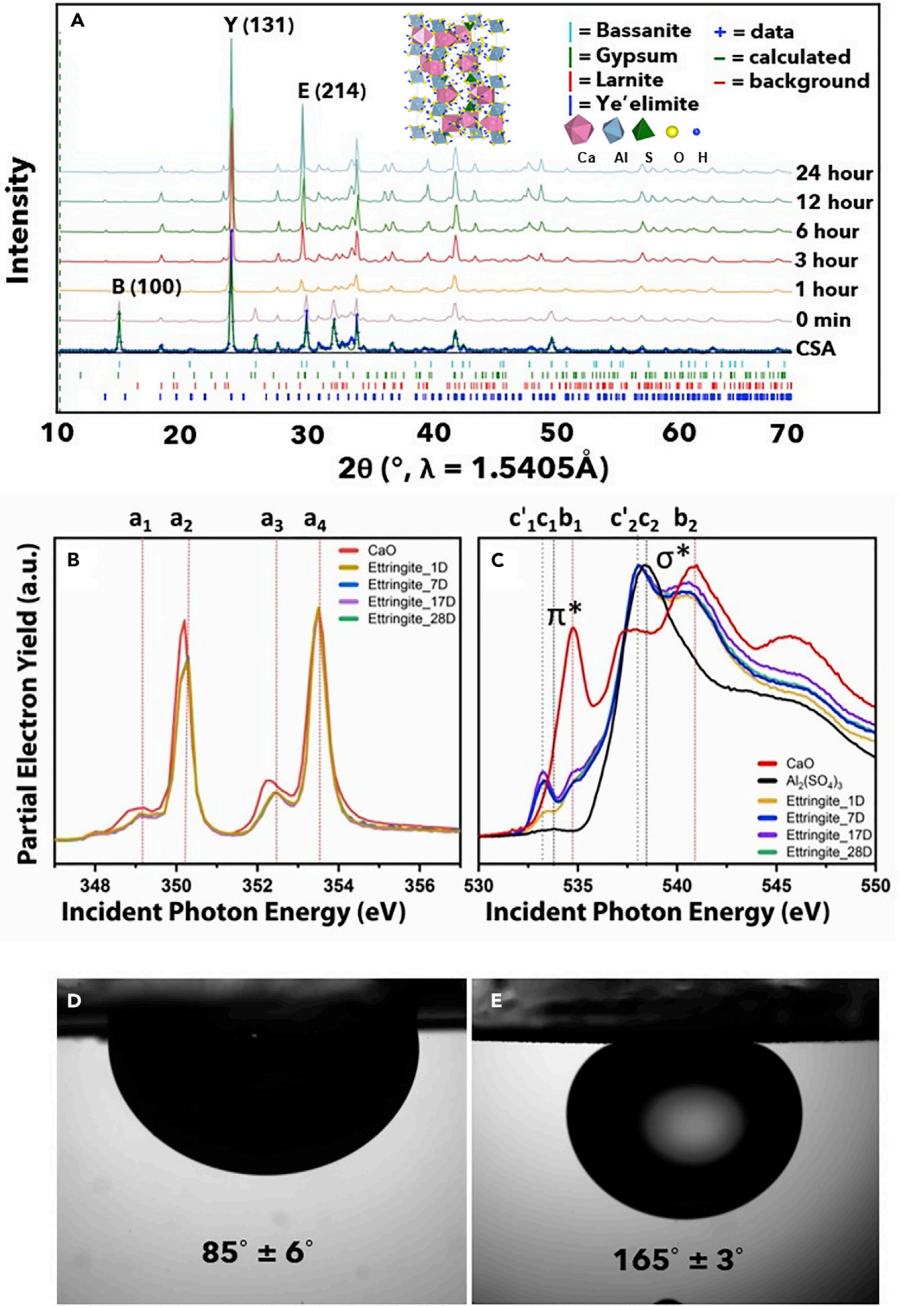
Wettability of CSA-based membranes, powder X-ray diffraction pattern (XRD), and X-ray Absorption Near Edge Structure (XANES) Characterization (A) Powder XRD patterns collected for CSA cement during hydration, with major refined phases indicated by tick marks. The pattern for the unreacted sample is labeled “CSA.” Patterns for samples after hydration lasting from 0 min (initial) to 24 h are shown from bottom to top, with a prominent (214) reflection for an Ettringite phase clearly evident after approximately 1 h of hydration. (Inset: crystal structure of ettringite. Pink and blue polyhedra represent oxygen-coordinated calcium- and aluminum-centered polyhedra, respectively). The inset shows a legend depicting the local coordination environments. (B) Normalized Ca L-edge XANES spectra for CaO and ettringite samples. (C) Normalized O K-edge XANES spectra for CaO, Al_2_(SO_4_)_3_, and ettringite samples. (D) Underwater contact angle of synthetic engine oil on bare stainless-steel mesh. (E) Underwater contact angle of synthetic engine oil on CSA/glass-sphere-coated membrane.

Power X-ray diffraction (XRD) patterns have been acquired to follow the hydration of CSA coatings within the membrane architectures (Figure 3A). After approximately 1 h, a fifth major phase appears in the XRD pattern that can be distinguished from the original components (calcium sulfoaluminate, calcium sulfate dihydrate, dicalcium silicate, calcium sulfate hemihydrate), and is indexed to hexacalcium aluminate trisulfate hydrate, designated as “ettringite” (the 214 reflection of ettringite is indicated by an asterisk in Figure 3A). Pawley fits have been used to evaluate the phase fractions with respect to the major refined phase, calcium sulfoaluminate, ye’elimite, (Figure S2) and are listed in Table S1 in the Supplemental Information.

The CSA coatings have been further examined by Ca L-edge XANES spectroscopy, which is compared to a CaO standard (Figure 3B). The coordination of Ca in tricalcium aluminate (3CaO⋅Al_2_O_3_) can be cubic or orthorhombic, whereas Ca is coordinated octahedrally in CSA hydrates such as kuzelite, gypsum, and ettringite (Geng et al., 2017). The measured XANES spectra are characterized by four prominent features. The doublets at Ca L_III_- and Ca L_II_-edges correspond to transitions from filled 2*p*_*3/2*_ and 2*p*_*1/2*_ core levels to unfilled states in the conduction band (Bae et al., 2016). Specifically, the doublets at the Ca L_III_- and Ca L_II_-edge arise from an asymmetric coordination environment present around the Ca^2+^-ion. The doublets a_1_ and a_2_ at the Ca L_III_-edge and a_3_ and a_4_ at the Ca L_II_-edge are ascribed to transitions into unoccupied states at the conduction band edge with t_*2g*_ and e_*g*_ symmetry. The relative energy positioning of these states is correlated with the strength of Ca—anion bonding; therefore, t_2g_ state is at higher energy than the e_g_ state when Ca is in cubic coordination and e_g_ state is at higher energy than the t_2g_ for octahedrally coordinated Ca. The Ca L-edge XANES spectrum of ettringite is similar to that of octahedrally coordinated Ca; the energy separation of *c.a.* 1.2 eV between a_1_-a_2_ and a_3_-a_4_ doublets is furthermore consistent with previous analyses of ettringite (Rajendran et al., 2013). The results thus provide corroboration for the ettringite crystal structure being an accurate depiction of the local structure of the calcium species.

O K-edge XANES spectra have further been acquired and are compared to spectra measured for CaO and Al_2_(SO_4_)_3_ standards as shown in Figure 3C. In the ettringite samples, several crystallographically inequivalent oxygen atoms are bonded in a multitude of chemical environments to S, Ca, and Al atoms. The different oxygen coordination environments yield a complex electronic structure which is manifested as multiple spectroscopic features in O K-edge XANES spectra. These spectroscopic features as shown in Figure 3C can be broadly divided into two categories. The features from 530 to 536 eV can be attributed to electronic excitations from O 1s core-levels to π∗ states (O 1s—π∗, such as Al-O and S-O hybrid states in Al_2_(SO_4_)_3_), whereas the broad features starting around 537 eV can be assigned to the electronic transitions of O 1s core-level electrons to electronic states with a Ϭ∗ character (e.g., S-O Ϭ∗electronic states in Al_2_(SO_4_)_3_) (Frati et al., 2020; Henderson et al., 2009). Using CaO and Al_2_(SO_4_)_3_ O K-edge XANES spectra as standards, the major features observed in the O K-edge XANES spectrum of (Ca_6_Al_2_(SO_4_)_3_(OH)_12_.26H_2_O) can be duly assigned. The feature labeled c'_1_ derives from the excitation of O 1s core-level electrons to S-O and Al-O states with a π∗ character. The increased intensity of this feature with respect to Al_2_(SO_4_)_3_ can be rationalized based on the presence of [Al(OH)_6_]^3−^ polyhedral units in the ettringite structure (Goetz-Neunhoeffer and Neubauer, 2006). The feature at 534.7 eV is attributed to electronic excitations into Ca—O π∗ states. The feature labeled c'_2_ arises from the excitation of O1*s* core-level electrons to Al—O and S—O Ϭ∗electronic states. The broad feature at 540.8 eV can be attributed to the excitation of core-level electrons to Ϭ∗ states resulting from Ca—O interactions. Overall, all four ettringite samples show similar spectroscopic features; therefore, both the Ca L-edge and O K-edge local structure probes provide evidence for ettringite formation across the four representative samples. Ca L- and O K-edge spectra for ettringite samples after 1, 7, 17, and 28 days show similar spectroscopic features, which attest to the early setting of ettringite, with scarce little modification of the local coordination environment after 1 day.

We next turn our attention to measurements of functional properties. Emulating the behavior of fish scales and the submerged surfaces of lotus leaves, underwater superoleophobic surfaces hold promise for breaking oil-in-water emulsions. Designing superhydrophilic and underwater superoleophobic surfaces are challenging as the surfaces should possess surface energy greater than water and less than oil (Zarghami et al., 2019). This requires a combination of hierarchically micro-/nanotextured materials and the incorporation of hydrophilic materials with enhanced surface roughness. The wettability of the CSA-based membranes has been measured by adapting a pendant drop-deposition method for liquid-submerged low-energy surfaces (Waghmare et al., 2013). In this approach, a low-energy substrate is submerged in a water-filled cuvette; a drop of oil is dispensed onto the submerged substrate using a needle. Contact angles are measured after allowing the oil droplets to settle on the surface. Figures 3D, 3E depict contact angle measurements for synthetic engine oil (SAE viscosity grade 5W-40) droplets on bare stainless-steel mesh substrate (pore size of 5 μm) and a CSA/glass-sphere-coated membrane. As a result of the 3D texturation evidenced in Figure 2, the coated membrane surface exhibits an oil contact angle of 165 ± 3°, unambigiously within a superoleophobic regime. Video S1 in the Supplemental Information displays demonstrates that when a water droplet is placed in contact with the coated membrane, it flash-spreads within just a few milliseconds to a contact angle of 0°, demonstrating a pronounced hydrophilic character. As such, the textured composite membranes exhibit a distinctive combination of underwater superhydrophilic and superoleophobic character, which we have sought to exploit to separate oil-in-water emulsions.

Video S1. Illustration of the hydrophilic behavior of the cement-based membranes, related to Figure 3 (D and E)

### Navigation of design space to optimize calcium sulphoaluminate/glass spheres membranes for desilting and deoiling produced water

Figure S3 illustrates a map of the Permian Basin, indicating the locations of 14 different wells from which PW samples have been collected. Wells 1-5 are in the Northern Midland sub-basin, Wells 6-10 in Southern Midland, and Wells 11-14 in the Delaware Basin.

**Figure 4:**
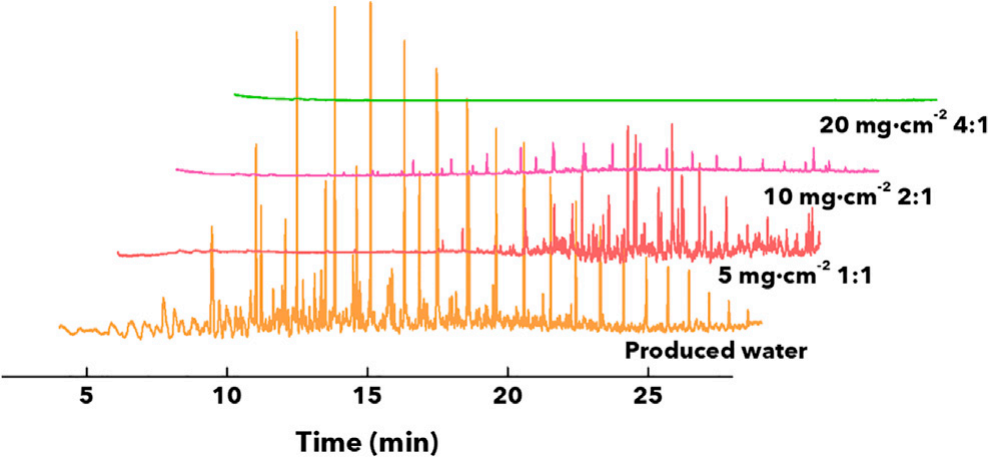
Gas chromatography-mass spectrometry (GC-MS) analysis of PW and filtered streams collected for CSA/GS-coated membranes Comparison of GC-MS for produced water, and CSA/GS-coated stainless-steel mesh membranes: (1) loading of 5 mg cm^−2^ at a CSA/GS ratio of 1:1; (2) loading of 10 mg cm^−2^ at a CSA/GS ratio of 2:1; and (3) loading of 20 mg cm^−2^ at a CSA/GS ratio of 4:1.

A membrane module as sketched in Figure S4 of the Supplemental Information is used to affect PW deoiling and desilting (Molecule Works Inc., 2020). CSA/glass-sphere-coated stainless steel mesh membranes are sandwiched between stainless-steel sweep and feed flow plates. The PW flows through the system at flux rates controlled by a peristaltic pump. We have evaluated the separation performance of the CSA-based membrane for PW collected from all 14 wells. Table S2 in the Supplemental Information displays the separation performance for each of the collected samples. Clear evidence for desilting is observed even at a flux rate of 1600 L h^−1^۰m^−2^. A systematic evaluation of membrane performance as a function of coating thickness and porosity has been performed using PW sampled from Well 9, in the Southern Midland region. Gas chromatography-mass spectrometry and turbidity measurements have been used to evaluate the efficacy of separation achieved by coated membranes in deoiling and desilting, respectively. Table S3 in the Supplemental Information shows the oil fractions detected in PW from Well 9 along with their corresponding retention times and molecular weight. The detected hydrocarbon fractions range from C11 to C30, with molecular weights ranging from 156 to 453 g/mol. Membrane architectures with different weight ratios of CSA cement and GS (4:1, 2:1, 1:1), as well as different overall loadings of CSA/GS (5, 10, 15, and 20 mg cm^−2^; correlated with coating thickness), have been evaluated for their efficacy in desilting and deoiling PW. Figure 4 contrasts chromatograms for PW obtained from Well 9, alongside chromatograms for permeates filtered through membranes with three representative coating formulations and thicknesses. Increasing the overall CSA/GS loading engenders greater tortuosity (as demonstrated in Figure 2), resulting in a substantial diminution of oil in the filtered samples. Indeed, at a CSA/GS ratio of 4:1 and total loading of 20 mg cm^−2^, no hydrocarbons are observed in the filtered stream within instrumental detection limits.

Detailed quantitative analyses of oil content in the filtered water have been performed for membrane formulations systematically varying the coating thickness and weight ratio of CSA to glass spheres. Calibration curves illustrated in Figure S5 were utilized to determine the concentration of hydrocarbons present in the PW and filtered water samples. Table S4 in the Supplemental Information summarizes the oil concentrations (in ppb) alongside their corresponding separation efficiency for the most abundant hydrocarbon fractions detected in the filtered water for each coating formulation.

**Figure 5:**
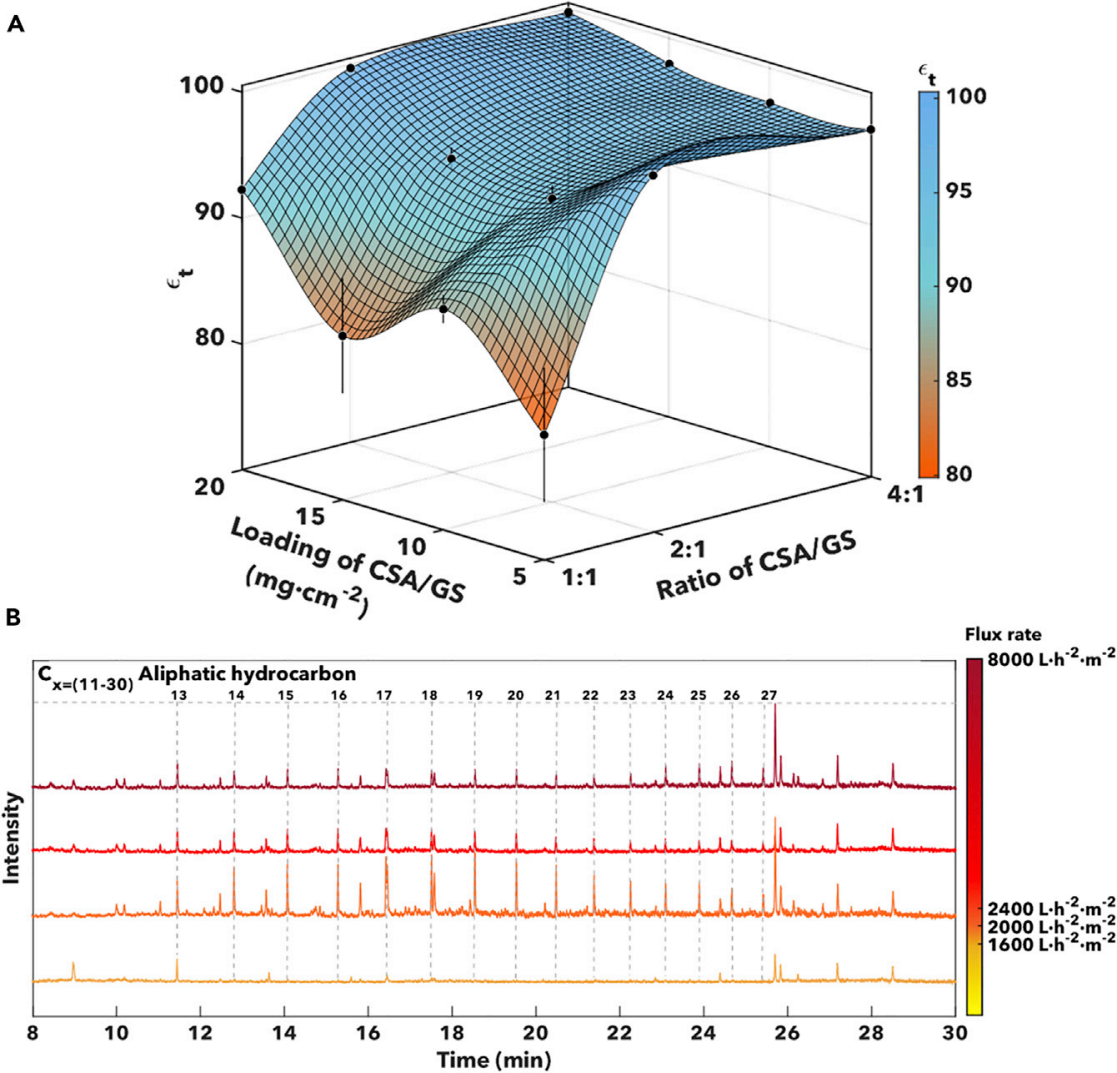
Evaluation of membrane performance across design space of weight ratio of CSA:glass spheres, coating thickness, and flux rate (A) Flux-rate-dependent GC-MS results for PW from Well 9 considering rates from 1600 to 8000 L h^−1^۰m^−2^ (using a CSA/glass-sphere-coated membrane with the loading of 20 mg cm^−2^ at a CSA/GS ratio of 4:1). Error bars represent the standard deviation of measurements run in triplicates. (B) 3D plot of total separation efficiency considering all aliphatic hydrocarbons present in filtered water as a function of CSA/GS loading (in mg·cm^−2^) and the ratio of CSA/GS and flux rate (1600-8000 L h^−1^۰m^−2^). Coating thicknesses ranging from 55 ± 10 μm to 207 ± 35 μm and loading from 5 to 20 mg cm^−2^ were evaluated.

At a CSA/GS loading of 5 mg cm^−2^, the highest detectable oil concentration was 18.30 ± 0.09 ppb (from C22), which corresponds to a separation efficiency of 41.0% for a CSA/GS weight ratio of 1:1. In contrast, increasing the proportion of CSA to 4:1 result in an oil concentration of only ca. 1.40 ± 0.02 ppb in the permeate (from C23), which represents a 94.5% separation efficiency. At a CSA/GS loading of 10 mg cm^−2^, the highest detectable oil concentration was 26.40 ± 0.40 ppb (from C22), rendering a separation efficiency of 14.9%, and the lowest was 1.45 ± 0.07 ppb (from C23) with a 94.3% of separation efficiency when the CSA content was increased to a weight ratio of 4:1. The rise in CSA concentration promoted the approximately 79% increase in the separation efficiency in the individual hydrocarbon fractions. At a CSA/GS loading of 15 mg cm^−2^, the highest detectable oil concentration was 18.31 ± 6.76 ppb (from C22), which represents a separation efficiency of 40.9%, and the lowest was 1.24 ± 0.01 ppb (from C22) corresponding to a 96.0% of separation efficiency. At a CSA/GS loading of 20 mg cm^−2^, the highest detectable oil concentration was 1.43 ± 0.003 ppb (from C18), which represents a separation efficiency of 96.7%, and the lowest was 1.28 ± 0.03 ppb (from C13) corresponding to a 98.8% of separation efficiency. Moreover, as the loading of CSA/GS is increased, corresponding to increasing coating thickness, the concentration of oil in the permeate is successively decreased, and an increasing number of hydrocarbon fractions are undetectable (see also Table S4 in the Supplemental Information). At a total CSA/GS loading of 20 mg cm^−2^ and weight ratios of CSA/GS of 2:1 and 4:1, the only detectable oil fraction corresponds to C13 (MW of 184 g/mol, Table S3). For each of the membrane architectures evaluated, we determined the overall separation efficiency (depicted in Figure 5A and determined using Equations (Equation 1), (Equation 2), (Equation 3) in Method details section) considering the concentration of oil for all the aliphatic hydrocarbon fractions present in the PW samples in comparison to the filtrated water samples. The total separation efficiency parameter was correlated with the CSA/GS total loading and CSA/GS weight ratios. The total separation efficiency values for CSA/GS total loadings of 20 mg cm^−2^ at CSA/GS ratios of 2:1 and 4:1 were 99.7 ± 0.01%. These results have allowed us to converge on 20 mg cm^−2^ CSA/GS total loadings at CSA/GS ratios of 2:1 and 4:1 as the most optimal coatings for deoiling of PW. A control experiment has been performed using a coating solely comprising CSA without any glass beads. Table S5 in the Supplemental Information tabulates measured oil concentrations in the permeate detected after single-pass filtration of 0.5 and 1 L of PW. It is clear that the composite membrane incorporating glass spheres shows appreciably better performance for different hydrocarbon fractions. The glass spheres add to the 3D texturation of the membrane as observed in Figure 2, and thus play a pivotal role in enhancing oil removal. Figure 5A shows that with increasing CSA content in the formulation, there is an increase in separation (deoiling) efficiency. As control experiments, we have evaluated meshes i) embedding only CSA cement without glass beads, ii) membranes comprising separately grown ettringite needles (Baur et al., 2004). In the first case, although we have a better adhesion of the cement phase owing to *in-situ* hydration on the membrane surface that is responsible for crosslinking the crosslinked-network couldn’t sustain the operational fluxes and hence we see the degradation in the quality of filtrate (Table S5) owing to the absence of textural effects afforded by glass spheres. In the case of the latter, as the ettringite needles are first grown in solution and then sprayed onto the surface to achieve high surface roughness, adhesion is compromised owing to the lack of *in-situ* hydration that results in crosslinked networks to the substrate.

The flux rate represents a key parameter in membrane separation technologies (O’Loughlin et al., 2018). Flux-rate-dependent separation analyses (Figure 5B) have been performed for optimal membrane modules (CSA/GS ratio of 4:1, CSA/GS loading of 20 mg cm^−2^) at flux rates ranging from 1600 to 8000 L h^−1^۰m^−2^. Flux rates below 1600 L h^−1^۰m^−2^ were not evaluated as a result of the unsteady flow observed. With the increasing flux rate from 2000 to 8000 L h^−1^۰m^−2^, Figure 5B shows increasing concentrations of C13 to C27 hydrocarbons in the permeate. At the lower flux rate (i.e., 1600 L h^−1^۰m^−2^), the only hydrocarbon fraction detected for the optimal membrane module (CSA/GS ratio of 4:1, CSA/GS loading of 20 mg cm^−2^) was C13, which furthermore appears at a very low intensity in the chromatogram. As such, the optimal flux rate has been found to be 1600 L h^−1^۰m^−2^, which corresponds to a value of ca. 10,000 gal·day^−1^۰m^−2^. As such, the membrane module offers the potential to clean large volumes of PW generated each day as necessary for industrial applications.

Figure S6 displays the results of turbidity measurements as a function of CSA/GS loading for membranes with different CSA/GS ratios, 1:1, 2:1, and 4:1, at varying flux rates. Figure S7-A in the Supplemental Information abstracts these results and plots turbidity as a function of the CSA/GS weight ratio and flux rate. At a constant CSA/GS weight ratio, the turbidity of the permeate decreases with decreasing flow rate. At a constant flux rate, the turbidity decreases with the amount of CSA. As such, optimal desilting is obtained at a flux rate of 1600 L h^−1^۰m^−2^ and high CSA content, corresponding to a CSA/GS weight ratio of 4:1. This also corresponds to optimal conditions for deoiling as shown in Figure S7-B in the Supplemental Information. This comprehensive analysis allows us to converge on a CSA coating with a relatively higher CSA to GS ratio and overall loading for optimal performance in desilting and deoiling of PW. High overall loadings translate to greater membrane tortuosities, which affords opportunities for the retention of silt particles and rejection of oil droplets at the superoleophobic and hydrophilic solid/liquid interface. Under these membrane conditions, the oil content in the filtered water is decreased to as low as 1 ppb with an overall separation efficiency of 99.7%, and indeed several oil fractions from C14 to C23 (MW of 198—324 g/mol) are no longer detectable in the permeate at single pass filtration.

**Figure 6:**
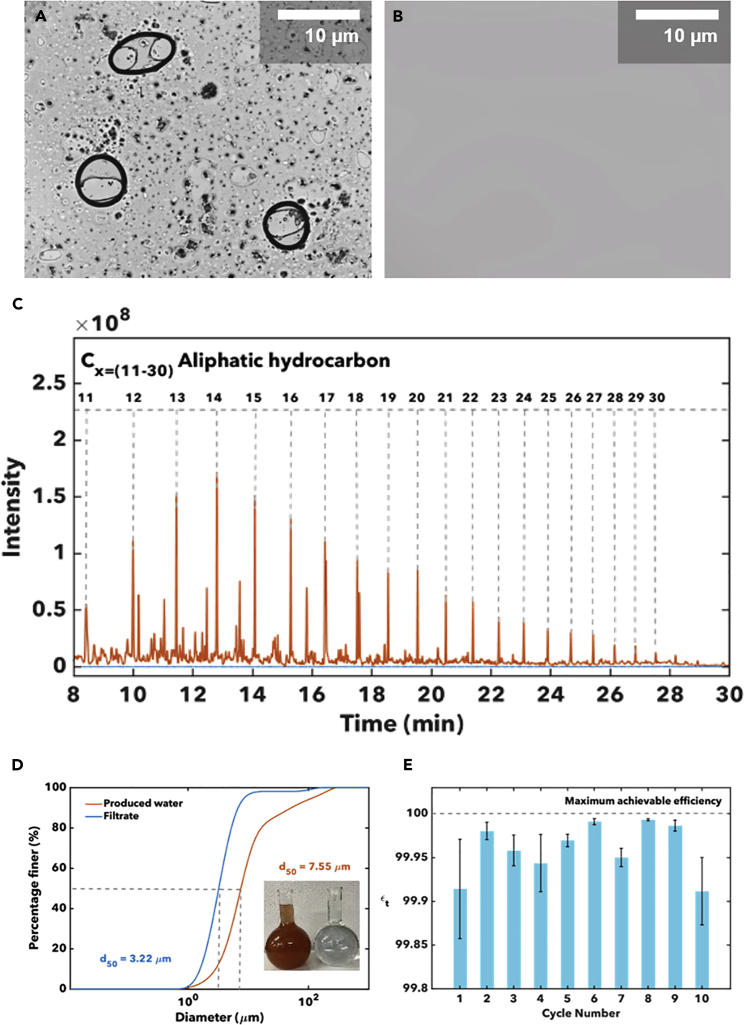
Evaluation of Membrane Module with an Optimal CSA/GS Coating (A) Optical microscopy image of PW emulsion from Well 9. Scale bars, 10 μm. (B) Optical microscopy image of filtered water recovered from optimal membrane module (CSA/GS ratio of 4:1, CSA/GS loading of 20 mg cm^−2^). Scale bars, 10 μm. (C) GC-MS showing the aliphatic hydrocarbons present in the PW emulsion from Well 9 and the corresponding chromatogram for filtered water collected from the membrane module. (D) Particle size analysis for PW from well 9 in the Permian Basin and filtered water recovered after separation. The digital photograph in the inset illustrates the visual change. (E) The separation efficiency of the CSA-based membrane determined by the oil concentration after 10 separation cycles and backwash. Error bars represent the standard deviation of measurements run in triplicates.

Figures 6A and 6B contrast optical microscopy images of PW from Well 9 and filtered water collected from the optimal membrane module with a CSA/GS ratio of 4:1 and a CSA/GS loading of 20 mg cm^−2^. In Figure 6A, oil droplets and silt particles are clearly visible within the continuous aqueous phase. Figure 6B shows that these are no longer discernible in the filtered permeate. Figure 6C provides a direct comparison of GC-MS results for Well 9 and the filtered water collected after passing through a membrane module with the above-mentioned optimal coating. No C11—C30 hydrocarbons are observable within instrumental detection limits. In addition to the sharp decrease in the concentration of silt particles measured by turbidimetry, particle size analysis has further been performed to examine the dimensions of permeated particles (Figure 6D). Although the particle size distribution is centered around 7.55 μm in PW, the average particle size in the permeate is much smaller at 3.22 μm reflecting the retention of relatively larger particles on the membrane surface.

Figures S8A and S8B in the Supplemental Information shows digital photographs of a membrane with the optimal coating formulation before and after the filtration of 0.5 L of PW. Silt and oil are collected at the surface. The coated membrane is able to sustain 10 cycles of backwashing with little to no degradation of the coating under operational conditions (Figures S8C and S8D in the Supplemental Information). Figure 6E shows the separation efficiency measured through 10 cycles for the membrane with a CSA/GS ratio of 4:1, CSA/GS loading of 20 mg cm^−2^. In each cycle, 50 mL of PW water were passed through the membrane module and backwashing was conducted with ca. 5 mL of clean water to remove the retained silt and oil. No additional chemicals were added during the backwashing cycles. The separation efficiency remains constant at ca. 99% within measurement error across the 10 cycles. Figures S8C and S8D in the Supplemental Information depict the membrane surface and residue after the first and tenth cycles. The membrane architecture is retained without evidence for delamination and with no loss of separation efficiency. SEM and powder XRD patterns were acquired for the recovered membrane after separation and backwashing steps, and are shown in Figures S9 and S10, respectively. In comparing the SEM images of a pristine membrane (e.g., Figure S9A) and a membrane after initial separation (e.g., Figure S9B), the latter shows the presence of entrained silt particles. After backwashing with 5 mL of water (e.g., Figure S9C), the preservation of the ettringite needle framework is evident in SEM images. The XRD pattern for backwashed membranes further attests to the preservation of ettringite, corroborating the robustness of the membrane modules.

Mercury porosimetry characterization was performed for as-prepared membranes after 24 h hydration, after the initial separation of a produced water stream, and after a backwash cycle. The total pore area, porosity, and tortuosity for an as-prepared membrane were 52 m^2^ g, 74%, and 3.4, respectively. The total pore area, porosity, and tortuosity after an initial separation step were 43 m^2^ g, 52%, and 13.5, respectively, which is consistent with the entrainment of silt particles. The total pore area, porosity, and tortuosity after backwash were 39 m^2^ g, 64%, and 3.8, respectively. As such, even though the pore area is reduced, the porosity and tortuosity are substantially recovered after backwashing, which enables further separation and recyclability of the membrane module.

The membrane modules developed here based on low-cost and earth-abundant materials show exceptional performance for the separation of recalcitrant emulsions without any pre-treatment and afford an intriguing combinational of mechanical resilience under high pressures, sustain high flow rates compatible with the industrial operation, and demonstrate exceptional selectivity. A clear correlation is observed between surface texturation, tortuosity, interfacial wettability, and separation performance in the separation of complex emulsions stabilized by endogenous surfactants. The design and performance of the membrane module presented in this work represent a new avenue for the separation of recalcitrant emulsions and are compared to current state-of-the-art membranes in Table S6.

### Conclusions

A robust membrane architecture has been developed for the treatment of PW in the Permian Basin, which exploits the underwater orthogonal wettability of ettringite needles formed by the hydration of CSA cement arrayed alongside glass spheres on stainless steel mesh substrates. The membranes show a combination of underwater superhydrophilic and superoleophobic behavior, which yields an exceptional combination of high selectivity, high flow rates, and effective retention of suspended solids. Oil concentration in PW is reduced to ca. ≤ 1 ppb, whereas turbidity resulting from silt particles is decreased by 2 orders of magnitude.

The membrane architecture comprises ettringite needles and glass spheres, which together define a 3D hierarchically textured surface with complex pore geometries that engender orthogonal wettability and result in high single-pass separation efficiency without the need for pre-treatment. Separation metrics have been examined as a function of membrane thickness, CSA:glass sphere weight ratios, and flux rates enabling rapid convergence on an optimal membrane geometry and flow conditions. The membranes are compact, mechanically resilient, readily cleanable through water backwash, and show near complete retention of functional performance. The results demonstrate a simple, scalable, and low-cost membrane module system which does not require pre-treatment, as is often necessary for current produced water treatment trains. The results further demonstrate the utility of a mechanism-informed approach to efficiently sample the materials design space to converge on an efficient separation system that affords a desired combination of functional performance parameters based on the optimization of 3D texturation and surface adsorption. The developed system offers new avenues for cleaning produced water and addressing emerging water scarcity concerns. The results demonstrated here for a single-pass system set the stage for process intensification through deployment within more sophisticated multi-pass membrane modules with extended interfaces and improved control of circulation parameters and flow patterns, as well as opportunities to dynamically control the feed flow rate, system temperature, and pressure (Alsarayreh et al., 2021; Venkatesan and Wankat, 2017). Future work will furthermore focus on coupling the membrane modules with solar desalination units to deliver compact water-treatment solutions for oil field use in the Permian Basin.

### Limitations of the study

There is intense interest in the cleaning of PW given the increasing worldwide focus on the development of unconventional assets; however, there is considerable variability in the composition of PW from region to region and as a function of different types of extractive processes. The membrane module designed in this study has been optimized for applications in the Permian Basin; however, the experimental approach is expected to be more generally applicable to PW in other regions. It is worth noting that a detailed accounting of all contaminants in PW is seldom available. As such, while the fundamental science reported here has broad applicability, the specific technology reported here is a singular illustration of a use case.

## STAR★Methods

### Key resources table

**Table:** 

REAGENT or RESOURCE	SOURCE	IDENTIFIER
**Chemicals, peptides, and recombinant proteins**		

Calcium sulfoaluminate cement (d_50_ < 11.89 μm (type V))	Silica Systems Inc.	CEMCSABUZ50LBS
Glass spheres, O_2_Si, particle size: 9–13 μm	Millipore Sigma	440345, CAS: 65997-17-3
Dichloromethane	EMD Millipore	DX0835, CAS: 75-09-2
Ethanol – 200 Proof	Koptec	Cat#: V1001G, CAS: 64-17-5
C8-C40 Alkanes Calibration Standards in Dichloromethane	Millipore Sigma	40147-U
Nitric Acid – Aqueous OmniTrace, 67–70%	Millipore Sigma	NX0407

**Software and algorithms**		

Thermo Xcalibur	Thermo Fisher Scientific	https://www.thermofisher.com/order/catalog/product/OPTON-30965?SID=srch-srp-OPTON-30965

**Other**		

MemXcel unit with stainless steel body	Molecule Works	MX-1-SS, https://www.moleculeworks.com/device (Molecule Works Inc., 2020)
Masterflex L/S Digital Drive with Easy-Load II Pump Head	Cole-Parmer	https://www.coleparmer.com/i/masterflex-l-s-digital-drive-with-easy-load-ii-pump-head-for-high-performance-tubing-115-230-vac/7792175 (Cole-Parmer, 2022)
2100Q Portable Turbidimeter	Hach	https://www.hach.com/2100q-portable-turbidimeter/product?id=7640450963 (Hach, 2022)

### Resource availability

#### Lead contact

Further information and requests for resources and reagents should be directed to and will be fulfilled by the lead contact, Sarbajit Banerjee (banerjee@chem.tamu.edu).

#### Materials availability

This study did not generate new unique reagents.

### Method details

#### Materials

Reagents and their commercial sources are as follows: CSA cement (Silica Systems Inc.); Dichloromethane (DCM, EMD Millipore); ethanol – 200 proof pure ethanol (Koptec); glass spheres: 9–13 μm particles, nitric acid – aqueous OmniTrace, and C8-C40 alkanes calibration standard in DCM (Millipore Sigma). All chemicals and solvents were used as received.

#### Membrane design: Incorporation of CSA and glass spheres onto stainless steel mesh

The main components of the membrane architecture include CSA, glass spheres, and stainless-steel mesh. In assembling the membrane, the CSA and glass sphere particles were dispersed in deionized water by ultrasonication (Branson Ultrasonic Bath 5510, Branson Ultrasonic Corp.) for ca. 5 min. The CSA-based dispersion was spray-coated onto a 304 stainless steel mesh (50 cm^2^, pore size: 5 μm, McMaster-Carr) using a master airbrush (0.5 mm nozzle diameter) coupled to an air compressor with a pressure of ca. 25 psi. During the spray-coating process, the substrate was heated to 200°C to aid water evaporation. After cooling to room temperature, the membrane was immersed in a water bath held at 25°C for 24 h to promote hydration of CSA. Different loadings (5, 10, 15, and 20 mg cm^−2^) of CSA/glass spheres and weight ratios of CSA to GS (4:1, 2:1, and 1:1) were evaluated to evaluate the optimal conditions for removal of silt and oil from produced water. This straightforward design based on a needle-like framework that reflects the intrinsic crystal growth of ettringite is readily scalable and show potential for large scale separation systems for recalcitrant emulsions (Figure S11 shows a 6ʹʹ×6ʹʹ CSA/GS-based membrane as an exemplar of scalable manufacturing).

#### Wettability of CSA-based membranes

Oil contact angles were measured using a CAM 200 Optical Goniometer. Synthetic engine oil (SAE Viscosity grade 5W-40, McMaster-Carr) and water were used to evaluate the superoleophobicity and superhydrophilicity of the membranes. For underwater oil contact angle measurements, the underwater contact angle was determined using a transparent quartz cuvette, a custom-made T-shaped steel substrate holder (304 stainless steel sheet with a size of 1 in × 1 in, McMaster-Carr, alongside a stainless steel rod), and an inverted “J” stainless steel needle with a 1 mL glass syringe (Waghmare et al., 2013; Liu et al., 2016). In the sessile drop measurements, the cuvette was filled with water, and the membrane was attached to the flat surface of the steel holder, which was positioned upside down so the substrate became submerged underwater while the steel rod was fixed to the goniometer. The membrane substrate was submerged into the water using a steel holder; next, a syringe was introduced to deliver ca. 5 μL oil droplets, which floated to the top to the membrane surface. Contact angle measurements were implemented in triplicate; ImageJ was used for analysis.

#### Characterization of membrane architecture

The hydration of CSA was tracked by powder X-ray diffraction (XRD) using a Bruker D8 Advance Eco X-ray powder diffractometer coupled with a Lynxeye detector (25 kV, 40 mA) and a Cu Kα (λ = 1.5418 Å) source. The morphology of the functionalized CSA-based membrane surface was imaged using a JEOL JSM-7500F field-emission scanning electron microscope coupled with a high brightness conical FE gun and a low-aberration conical objective lens. All samples for SEM imaging were coated with an ultra-thin platinum layer using a Cressington 208HR High-Resolution Sputter Coater.

Ca L- and O K- XANES measurements were performed at the National Synchrotron Light Source II of Brookhaven National Laboratory beamline SST-1 operated by the National Institute of Standards and Technology. Measurements were performed in partial electron yield (PEY) mode with a nominal resolution of 0.1 eV. The PEY signal was normalized to the incident beam intensity of a clean gold grid to eliminate the effects of any incident beam fluctuations and optics absorption features.

Mechanical testing of the CSA-GS coatings was implemented using ASTM D2197, where a U-shaped loop was placed to the surface and used to scrape the surface with added weights ranging from 0–300 g. These measurements provide a measure of coating adhesion. Samples for the mechanical testing were prepared on galvanized stainless-steel sheet substrates instead of membranes (6 in^2^).

#### Membrane module design: Separating silt and oil from PW

The membrane module system was built with a stainless-steel filtration cell, MemXcell (Model: MX-1-SS, Molecule Works Inc., Figure S4) (Molecule Works Inc., 2020), and connected to a peristaltic pump (Masterflex L/S Digital Drive with Easy-Load II Pump Head, 600 RPM, Cole-Palmer) with L/S 24 tubing (maximum pressure: 2.7 bar). The pressure of the membrane module is controlled by the tubing size, area of the filtration cell, and flux rates governed by the peristaltic pump. Fixed flow rates enable the dispensation of a certain volume of water at specific time intervals. In typical filtration experiments, the CSA-coated membrane (50 cm^2^) was positioned within the cell on the sweep plate and secured with two graphite gaskets; next, a feed flow plate was placed on the top and secured with screws. Before every run, the system was cleaned with hexanes, ethanol, powdered precision cleaner dilution (1:100 dilution, Alconox), and water (ca. 250 mL) at a flux rate of 1600 L h^−1^۰m^−2^. In each run, PW was flowed through the custom-designed system with the help of a peristaltic pump and the effluent stream was collected for further analysis. Flux rates ranging from 1600 to 8000 L h^−1^۰m^−2^ were analyzed to establish the optimal conditions for separating silt and oil from PW.

#### Performance evaluation of membranes

The presence of silt in the filtrate samples compared to the produced water was studied using turbidity measurements and particle size analysis. Turbidity measurements were collected by using a 2100Q portable turbidimeter. The turbidity measurements were calibrated using a Gelex secondary standards kit (10NT, 20, 100, 800 NTU). Immediately after collecting the filtrate, the turbidity measurements were performed to avoid sedimentation; six replicates were gathered for each sample.

The particle size distribution of silt particles in filtered samples was determined using a Horiba laser scattering particle size distribution analyzer (LA-960) with a size detection capacity ranging from 10 nm to 5 mm. Ethanol (ca. 180–250 mL, 99.5%) was used as a dispersion medium. Solid solutions of each sample type were used while performing at least three replicate measurements. For each run, samples were circulated for 2 min, agitated for 2 min, followed by ultrasonication for 2 min, and alignment and blanking of the instrument.

The presence of oil and silt in PW and filtrate streams was further evaluated using an Olympus BX41 optical microscope. The collected samples were pressed between two thin glass microscope slides. The samples were illuminated using a Euromex EK-1 halogen lamp fiber optic light source.

Mercury porosimetry characterization was performed to determine changes in membrane porosity and pore size distribution after water filtration. Porosity measurements were collected using a Micromeritics AutoPore IV 9510 instrument. Measurements were performed at MSE Supplies LLC. Sample weights ranging from ca. 1.5–2 g were used for the measurements. Samples evaluated were an as-prepared membrane after 24 h hydration, after initial separation of a produced water stream, and after a backwash cycle.

#### Quantitative separation analysis: Removal of silt and oil from produced water

The concentration of oil (in ppb) in PW and filtrate samples was examined by GC-MS using a Thermo Scientific DSQ II instrument. Data acquisition and processing were performed using Thermo Xcalibur software. Oil fractions ranging from C11 to C30 were detected in collected PW samples from across the Permian Basin (Figure S3). GC-MS samples were prepared by performing an extraction with a 6:1 (v/v) mixture of water and dichloromethane. Since water samples were collected in glass vials and oil can adhere to the glass wall of the vials, DCM was first added into the vials, and then the layers were mixed using a vortexer (Vortex-Genie 2) to collect all the oil. The two-phase mixture was then separated using a separation funnel and the decanted DCM layer was used for further analyses. Each sample was run in triplicate. Figure S5 in the Supplemental Information plots calibration curves for the most abundant oil fractions in the PW samples (i.e., C13—C23) generated using a C8—C40 Alkanes Calibration Standard. Standard solutions were prepared with concentrations ranging from 0.25–50 ng/mL in dichloromethane.

In assessing the stability of the membrane architecture throughout the separation process, the filtration system was run for ten cycles of running forward and backward. For each backwash, ca. 10% of water (out of the water used forward) was utilized to clean the membrane and restart the process. The elemental composition of the produced water and recovered produced water was determined using inductively coupled plasma-mass spectroscopy (PerkinElmer NexION 300D ICP-MS), and data collection and processing were conducted via Syngistix v2.4. Water samples (0.5 g) were digested in a 2% aqueous solution of HNO_3_ and diluted to 100 ng/mL.

#### Determination of the overall separation efficiency for deoiling in PW and filtered water samples

In order to effectively quantify the overall efficiency of separation ($\varepsilon_{t}$) of oil fractions from produced water, contribution of each aliphatic fraction ($C_{x}$) was normalized using Equation 1. This contribution was assigned as efficiency coefficient for that fraction ($eff_{C_{x}}$) and is characteristic of that fraction. Then, efficiency of each fraction was calculated using Equation 2. Effective efficiency of separation (includes contribution from all aliphatic fractions for a membrane composition) was calculated by summation of the product of the characteristic coefficient of efficiency for each aliphatic fraction and efficiency of that fraction (as depicted in Equation 3).

(Equation 1) $$Coefficient\;of\;eff_{C_{x}}\;=\;\frac{Concentration\;of\;C_{x}\;in\;PW}{Total\;concentration\;of\;aliphatic\;fractions\;in\;PW}$$

(Equation 2) $$eff_{C_{x}}=\;\frac{Concentration\;of\;C_{x}\;in\;PW-Concentration\;of\;C_{x}\;in\;filtrate}{Concentration\;of\;C_{x}\;in\;PW}\times 100$$

(Equation 3) $$\varepsilon_{t}=0.218\times eff_{C_{13}}+0.258\times eff_{C_{14}}+0.171\times eff_{C_{16}}+0.088\times eff_{C_{18}}+0.084\times eff_{C_{20}}+0.066\times eff_{C_{21}}+0.063\times eff_{C_{22}}+0.052\times eff_{C_{23}}$$

#### Determining the efficacy of CSA without glass spheres

Since higher concentrations of CSA yielded the most efficient separation, a control experiment was performed to examine the critical role of the glass spheres in the membrane architecture. Coated membranes were prepared using CSA particles only without glass spheres. In this “control” membrane, the CSA particles (loading of 20 mg cm^−2^) were spray-coated at 200°C onto a stainless-steel mesh (50 cm^2^), and the membrane was hydrated for 24 h. The resulting coated substrate was used within the membrane module. For each sample, 0.5 L of PW was filtered through the system. GC-MS and turbidity measurements were performed for the effluent streams to evaluate oil and silt concentrations.

## Data Availability

• All data reported in this paper will be shared by the lead contact upon request. • This paper does not report original code. • Any additional information required to reanalyze the data reported in this paper is available from the lead contact upon request.

## Supporting citations

Agi et al., 2018, Atallah et al., 2019, Atallah et al., 2017, Bigui et al., 2019, Chen et al., 2018, Kollarigowda et al., 2017, Kumar et al., 2015, Li et al., 2017a, Li et al., 2017b, Liu et al., 2019, Liu et al., 2020, Liu et al., 2017, Long et al., 2018, Mahdi et al., 2019, Ou et al., 2016, Wang et al., 2019, Wang et al., 2015, Xu et al., 2019, Yue et al., 2017, Yue et al., 2018, Zeng et al., 2017, Zhang et al., 2020, Zhang et al., 2013, Zhang et al., 2017, Zhang et al., 2018, Zhu et al., 2015.

## Supplemental information

Document S1. Figures S1–S11 and Tables S1–S5

Table S6. State-of-the-art membrane technologies for produced water treatment (Parts of the table were discussed in previous work (Douglas et al., 2022), related to Figure 1 and Figure 6)

## References

[bib1] Agi A., Junin R., Yahya A., Gbadamosi A., Abbas A. (2018). Comparative study of continuous and intermittent ultrasonic ultrafiltration membrane for treatment of synthetic produced water containing emulsion. Chem. Eng. Process. Process Intensif..

[bib2] Alammar A., Hardian R., Szekely G. (2022). Upcycling agricultural waste into membranes: from date seed biomass to oil and solvent-resistant nanofiltration. Green Chem..

[bib3] Alammar A., Park S.-H., Williams C.J., Derby B., Szekely G. (2020). Oil-in-water separation with graphene-based nanocomposite membranes for produced water treatment. J. Memb. Sci..

[bib4] Alsarayreh A.A., Al-Obaidi M.A., Farag S.K., Patel R., Mujtaba I.M. (2021). Performance evaluation of a medium-scale industrial reverse osmosis brackish water desalination plant with different brands of membranes. A simulation study. Desalination.

[bib5] Atallah C., Mortazavi S., Tremblay A.Y., Doiron A. (2019). Surface-Modified multi-lumen tubular membranes for SAGD-produced water treatment. Energy Fuels.

[bib6] Atallah C., Tremblay A.Y., Mortazavi S. (2017). Silane surface modified ceramic membranes for the treatment and recycling of SAGD produced water. J. Pet. Sci. Eng..

[bib7] Babayev M., Du H., Botlaguduru V.S.V., Kommalapati R.R. (2019). Zwitterion-Modified ultrafiltration membranes for Permian Basin produced water pretreatment. Water.

[bib8] Bae S., Kanematsu M., Hernández-Cruz D., Moon J., Kilcoyne D., Monteiro P.J.M. (2016). In situ soft X-ray spectromicroscopy of early tricalcium silicate hydration. Materials.

[bib9] Bajpayee A., Alivio T.E.G., McKay P., Banerjee S. (2019). Functionalized tetrapodal ZnO membranes exhibiting superoleophobic and superhydrophilic character for water/oil separation based on differential wettability. Energy Fuels.

[bib10] Baur I., Keller P., Mavrocordatos D., Wehrli B., Johnson C. (2004). Dissolution-precipitation behaviour of ettringite, monosulfate, and calcium silicate hydrate. Cem. Concr. Res..

[bib11] Bigui W., Cheng Y., Jianlin L., Gang W., Liang D., Xiaosan S., Fuping W., Hua L., Qing C. (2019). Fabrication of superhydrophilic and underwater superoleophobic quartz sand filter for oil/water separation. Sep. Purif. Technol..

[bib12] Bolto B., Zhang J., Wu X., Xie Z. (2020). A review on current development of membranes for oil removal from wastewaters. Membranes.

[bib13] Cai Y., Shi S.Q., Fang Z., Li J. (2021). Design, development, and outlook of superwettability membranes in oil/water emulsions separation. Adv. Mater. Interfaces.

[bib14] Chen J., Guo D., Huang C., Wen X., Xu S., Cheng J., Pi P. (2018). Fabrication of superhydrophobic copper mesh by depositing CuCl for oil/water separation. Mater. Lett..

[bib15] Chen T., Duan M., Fang S. (2016). Fabrication of novel superhydrophilic and underwater superoleophobic hierarchically structured ceramic membrane and its separation performance of oily wastewater. Ceram. Int..

[bib16] Cole-Parmer (2022). Masterflex L/S® digital drive with Easy-Load® II pump head for high-performance tubing; 115/230 VAC. https://www.coleparmer.com/i/masterflex-l-s-digital-drive-with-easy-load-ii-pump-head-for-high-performance-tubing-115-230-vac/7792175.

[bib17] Cooper C.M., McCall J., Stokes S.C., McKay C., Bentley M.J., Rosenblum J.S., Blewett T.A., Huang Z., Miara A., Talmadge M. (2021).

[bib18] Dickhout J.M., Moreno J., Biesheuvel P.M., Boels L., Lammertink R.G.H., de Vos W.M. (2017). Produced water treatment by membranes: a review from a colloidal perspective. J. Colloid Interface Sci..

[bib19] Douglas L.D., Rivera-Gonzalez N., Cool N., Bajpayee A., Udayakantha M., Liu G.-W., Anita, Banerjee S. (2022). A materials science perspective of midstream challenges in the utilization of heavy crude oil. ACS Omega.

[bib20] EIA (2021).

[bib21] Fakhru’l-Razi A., Pendashteh A., Abdullah L.C., Biak D.R.A., Madaeni S.S., Abidin Z.Z. (2009). Review of technologies for oil and gas produced water treatment. J. Hazard Mater..

[bib22] Frati F., Hunault M.O.J.Y., de Groot F.M.F. (2020). Oxygen K-edge X-ray absorption spectra. Chem. Rev..

[bib23] Geng G., Myers R.J., Kilcoyne A.L., Ha J., Monteiro P.J. (2017). Ca L_2, 3_-edge near edge X-ray absorption fine structure of tricalcium aluminate, gypsum, and calcium (sulfo)aluminate hydrates. Am. Mineral..

[bib24] Goetz-Neunhoeffer F., Neubauer J. (2006). Refined ettringite (Ca_6_Al_2_(SO_4_)_3_(OH)12·26H_2_O) structure for quantitative X-ray diffraction analysis. Powder Diffr..

[bib25] Hach (2022). 2100Q portable turbidimeter. https://www.hach.com/2100q-portable-turbidimeter/product?id=7640450963.

[bib26] Hargis C.W., Telesca A., Monteiro P.J. (2014). Calcium sulfoaluminate (Ye'elimite) hydration in the presence of gypsum, calcite, and vaterite. Cem. Concr. Res..

[bib27] Henderson G.S., Neuville D.R., Cormier L. (2009). An O K-edge XANES study of glasses and crystals in the CaO–Al_2_O_3_–SiO_2_ (CAS) system. Chem. Geol..

[bib28] Hussain F.A., Zamora J., Ferrer I.M., Kinyua M., Velázquez J.M. (2020). Adsorption of crude oil from crude oil–water emulsion by mesoporous hafnium oxide ceramics. Environ. Sci, Water Res. Technol..

[bib29] Igunnu E.T., Chen G.Z. (2012). Produced water treatment technologies. Int. J. Low-Carbon Tech..

[bib30] Jiménez S., Micó M.M., Arnaldos M., Medina F., Contreras S. (2018). State of the art of produced water treatment. Chemosphere.

[bib31] Kollarigowda R.H., Abraham S., Montemagno C.D. (2017). Antifouling cellulose hybrid biomembrane for effective oil/water separation. ACS Appl. Mater. Interfaces.

[bib32] Kumar S., Guria C., Mandal A. (2015). Synthesis, characterization and performance studies of polysulfone/bentonite nanoparticles mixed-matrix ultra-filtration membranes using oil field produced water. Sep. Purif. Technol..

[bib33] Li F., Yu Z., Shi H., Yang Q., Chen Q., Pan Y., Zeng G., Yan L. (2017). A Mussel-inspired method to fabricate reduced graphene oxide/g-C_3_N_4_ composites membranes for catalytic decomposition and oil-in-water emulsion separation. Chem. Eng. J..

[bib34] Li J., Zhao Z., Li D., Tian H., Zha F., Feng H., Guo L. (2017). Smart candle soot coated membranes for on-demand immiscible oil/water mixture and emulsion switchable separation. Nanoscale.

[bib35] Liu L., Chen C., Yang S., Xie H., Gong M., Xu X. (2016). Fabrication of superhydrophilic–underwater superoleophobic inorganic anti-corrosive membranes for high-efficiency oil/water separation. Phys. Chem. Chem. Phys..

[bib36] Liu S., Cai T., Shen X., Huang E., Wang Z., Sun Q. (2019). Superhydrophobic sand with multifunctionalities by TiO_2_-incorporated mussel-inspired polydopamine. Ceram. Int..

[bib37] Liu W., Cui M., Shen Y., Mu P., Yang Y., Li J. (2020). Efficient separation of crude oil-in-water emulsion based on a robust underwater superoleophobic titanium dioxide-coated mesh. New J. Chem..

[bib38] Liu Y., Su Y., Cao J., Guan J., Xu L., Zhang R., He M., Zhang Q., Fan L., Jiang Z. (2017). Synergy of the mechanical, antifouling and permeation properties of a carbon nanotube nanohybrid membrane for efficient oil/water separation. Nanoscale.

[bib39] Long Y., Shen Y., Tian H., Yang Y., Feng H., Li J. (2018). Superwettable Coprinus comatus coated membranes used toward the controllable separation of emulsified oil/water mixtures. J. Memb. Sci..

[bib40] Mahdi N., Kumar P., Goswami A., Perdicakis B., Shankar K., Sadrzadeh M. (2019). Robust polymer nanocomposite membranes incorporating discrete TiO_2_ nanotubes for water treatment. Nanomaterials.

[bib41] Mercelat A.Y., Cooper C.M., Kinney K.A., Seibert F., Katz L.E. (2021). Mechanisms for direct separation of oil from water with hydrophobic hollow fiber membrane contactors. ACS EST. Eng..

[bib42] Molecule Works Inc (2020). Miniature filter and separator design (MemXcel). https://moleculeworks.com/device.

[bib43] Neff J.M., Lee K., DeBlois E.M., Lee K., Neff J. (2011). Produced Water.

[bib44] O'Loughlin T.E., Martens S., Ren S.R., McKay P., Banerjee S. (2017). Orthogonal wettability of hierarchically textured metal meshes as a means of separating water/oil emulsions. Adv. Eng. Mater..

[bib45] O’Loughlin T.E., Ngamassi F.-E., McKay P., Banerjee S. (2018). Separation of viscous oil emulsions using three-dimensional nanotetrapodal ZnO membranes. Energy Fuels.

[bib46] Olajire A.A. (2020). Recent advances on the treatment technology of oil and gas produced water for sustainable energy industry-mechanistic aspects and process chemistry perspectives. Chem. Eng. J. Adv..

[bib47] Ou R., Wei J., Jiang L., Simon G.P., Wang H. (2016). Robust thermoresponsive polymer composite membrane with switchable superhydrophilicity and superhydrophobicity for efficient oil–water separation. Environ. Sci. Technol..

[bib48] Padaki M., Surya Murali R., Abdullah M.S., Misdan N., Moslehyani A., Kassim M.A., Hilal N., Ismail A.F. (2015). Membrane technology enhancement in oil–water separation. A review. Desalination.

[bib49] Rajendran J., Gialanella S., Aswath P.B. (2013). XANES analysis of dried and calcined bones. Mater. Sci. Eng. C Mater. Biol. Appl..

[bib50] Rodriguez A.Z., Wang H., Hu L., Zhang Y., Xu P. (2020). Treatment of produced water in the Permian Basin for hydraulic fracturing: comparison of different coagulation processes and innovative filter media. Water.

[bib51] Scanlon B.R., Ikonnikova S., Yang Q., Reedy R.C. (2020). Will water issues constrain oil and gas production in the United States?. Environ. Sci. Technol..

[bib52] Scanlon B.R., Reedy R.C., Male F., Walsh M. (2017). Water issues related to transitioning from conventional to unconventional oil production in the Permian Basin. Environ. Sci. Technol..

[bib53] Scanlon B.R., Reedy R.C., Wolaver B.D. (2021). Assessing cumulative water impacts from shale oil and gas production: Permian Basin case study. Sci. Total Environ..

[bib54] Scanlon B.R., Reedy R.C., Xu P., Engle M., Nicot J.P., Yoxtheimer D., Yang Q., Ikonnikova S. (2020). Can we beneficially reuse produced water from oil and gas extraction in the U.S.?. Sci. Total Environ..

[bib55] Venkatesan A., Wankat P.C. (2017). Produced water desalination: an exploratory study. Desalination.

[bib56] Waghmare P.R., Das S., Mitra S.K. (2013). Drop deposition on under-liquid low energy surfaces. Soft Matter.

[bib57] Wang H. (2020). The economic impact of oil and gas development in the Permian Basin: local and spillover effects. Resour. Policy.

[bib58] Wang H. (2021). Shale oil production and groundwater: what can we learn from produced water data?. PLoS One.

[bib59] Wang X., Li M., Shen Y., Yang Y., Feng H., Li J. (2019). Facile preparation of loess-coated membranes for multifunctional surfactant-stabilized oil-in-water emulsion separation. Green Chem..

[bib60] Wang Z., Jiang X., Cheng X., Lau C.H., Shao L. (2015). Mussel-inspired hybrid coatings that transform membrane hydrophobicity into high hydrophilicity and underwater superoleophobicity for oil-in-water emulsion separation. ACS Appl. Mater. Interfaces.

[bib61] Xu X., Long Y., Li Q., Li D., Mao D., Chen X., Chen Y. (2019). Modified cellulose membrane with good durability for effective oil-in-water emulsion treatment. J. Clean. Prod..

[bib62] Yue X., Li J., Zhang T., Qiu F., Yang D., Xue M. (2017). In situ one-step fabrication of durable superhydrophobic-superoleophilic cellulose/LDH membrane with hierarchical structure for efficiency oil/water separation. Chem. Eng. J..

[bib63] Yue X., Zhang T., Yang D., Qiu F., Li Z. (2018). Hybrid aerogels derived from banana peel and waste paper for efficient oil absorption and emulsion separation. J. Clean. Prod..

[bib64] Zarghami S., Mohammadi T., Sadrzadeh M., Van der Bruggen B. (2019). Superhydrophilic and underwater superoleophobic membranes - a review of synthesis methods. Prog. Polym. Sci..

[bib65] Zeng X., Qian L., Yuan X., Zhou C., Li Z., Cheng J., Xu S., Wang S., Pi P., Wen X. (2017). Inspired by stenocara beetles: from water collection to high-efficiency water-in-oil emulsion separation. ACS Nano.

[bib66] Zhang C., Zhang T., Huang J., Yan T., Li C., Liu L., Wang L., Jiao F. (2020). Copper hydroxyphosphate nanosheets-covered robust membranes with superhydrophilicity and underwater ultralow adhesive superoleophobicity for oil/water separation and visible light photodegradation. Colloids Surf. A Physicochem. Eng. Asp..

[bib67] Zhang F., Zhang W.B., Shi Z., Wang D., Jin J., Jiang L. (2013). Nanowire-haired inorganic membranes with superhydrophilicity and underwater ultralow adhesive superoleophobicity for high-efficiency oil/water separation. Adv. Mater..

[bib68] Zhang J., Xue Q., Pan X., Jin Y., Lu W., Ding D., Guo Q. (2017). Graphene oxide/polyacrylonitrile fiber hierarchical-structured membrane for ultra-fast microfiltration of oil-water emulsion. Chem. Eng. J..

[bib69] Zhang S., Jiang G., Gao S., Jin H., Zhu Y., Zhang F., Jin J. (2018). Cupric phosphate nanosheets-wrapped inorganic membranes with superhydrophilic and outstanding anticrude oil-fouling property for oil/water separation. ACS Nano.

[bib70] Zhou Q., Milestone N.B., Hayes M. (2006). An alternative to Portland Cement for waste encapsulation—the calcium sulfoaluminate cement system. J. Hazard Mater..

[bib71] Zhu Y., Xie W., Li J., Xing T., Jin J. (2015). pH-Induced non-fouling membrane for effective separation of oil-in-water emulsion. J. Memb. Sci..

[bib72] Zolghadr E., Firouzjaei M.D., Amouzandeh G., LeClair P., Elliott M. (2021). The role of membrane-based technologies in environmental treatment and reuse of produced water. Front. Environ. Sci..

[bib73] Zou D., Kim H.W., Jeon S.M., Lee Y.M. (2022). Robust PVDF/PSF hollow-fiber membranes modified with inorganic TiO_2_ particles for enhanced oil-water separation. J. Membr. Sci..

[bib74] Zoubeik M., Salama A., Henni A. (2019). A comprehensive experimental and artificial network investigation of the performance of an ultrafiltration titanium dioxide ceramic membrane: application in produced water treatment. Water Environ. J..

