# Supplementary material for: Textured ceramic membranes for desilting and deoiling of produced water in the Permian Basin
Source: iScience. 2022 Sep 5;25(10):105063. doi: 10.1016/j.isci.2022.105063 (PMC9490591; doi:10.1016/j.isci.2022.105063)
Supplement: Document S1. Figures S1–S11 and Tables S1–S5 [file mmc1.pdf]

iScience, Volume *25*

Supplemental information

Textured ceramic membranes for desilting and deoiling of produced water in the Permian Basin

Natalia Rivera-Gonzalez, Aayushi Bajpayee, Jakob Nielsen, Umme Zakira, Wasif Zaheer, Joseph Handy, Tiffany Sill, Bjorn Birgisson, Mukul Bhatia, and Sarbajit Banerjee

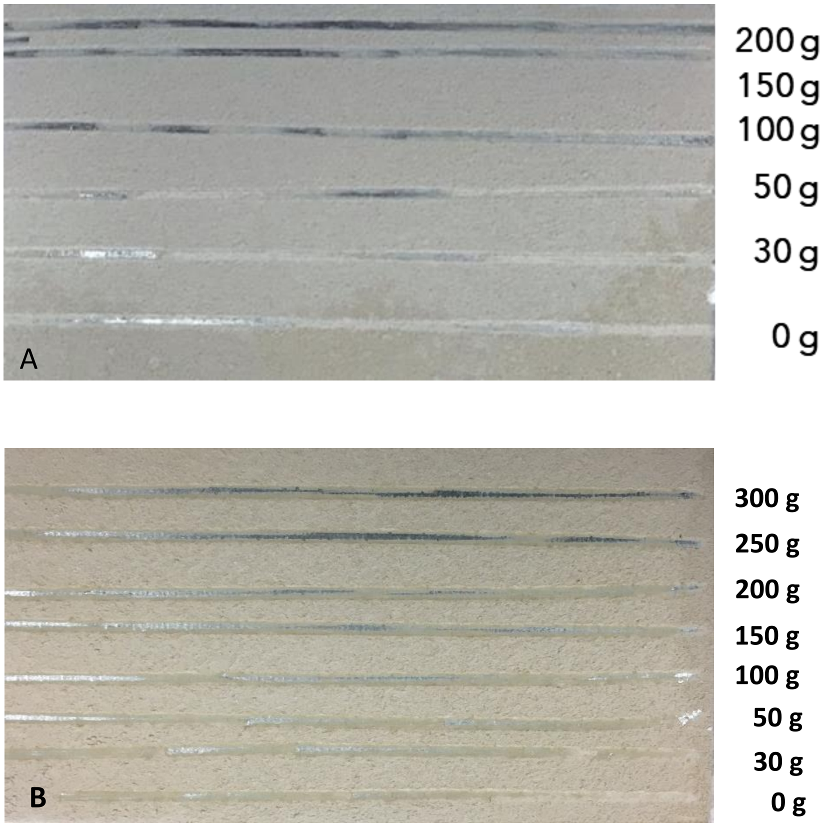

Figure S1. Digital photograph of CSA-based membrane under scrape test, ASTM 2197, related to Figure 1 and Figure 2.

(A) Hydration of CSA: 1 day sample

(B) Hydration of CSA: 28-day sample

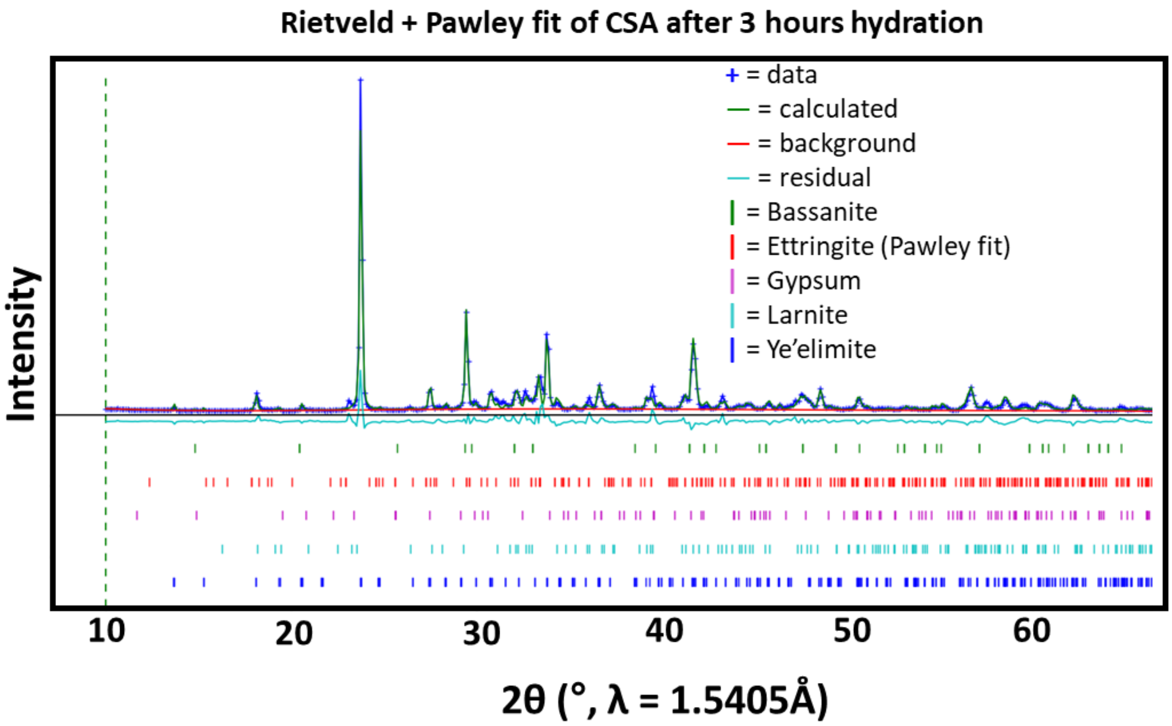

Figure S2. Powder XRD patterns for hydrated CSA, related to Figure 3A. Combined Rietveld + Pawley fit for powder XRD pattern acquired for a CSA sample after 3 h hydration (whole pattern residual R_w = 16.9%). Tick marks are shown for refined phases. Bassanite, Gypsum, Larnite, and Ye'elimite were refined using known atomic structures. Owing to intensity mismatch likely arising from variance in hydration, reflections from Ettringite were modeled using a Pawley fit to the lattice parameters for a known structure.

Table S1: Refined phase weight fractions relative to ye'elimite from Pawley fit of XRD data, related to Figure 3A and Figure S2.

	Refined Phase Weight Fractions Relative to Ye'elimite			
	**Ye'elimite**	**Larnite**	**Bassanite**	**Gypsum**
**Unhydrated CSA**	1	0.273	0.596	0.026
**0 min**	1	0.288	0.569	0.023
**10 min**	1	0.299	0.031	0.022
**30 min**	1	0.281	0.031	0.039
**1 h**	1	0.315	0.027	0.055
**3 h**	1	0.230	0.029	0.001
**6 h**	1	0.157	0.025	0.039
**12 h**	1	0.080	0.032	0.052
**24 h**	1	0.053	0.033	0.053
**48 h**	1	0.065	0.036	0.089

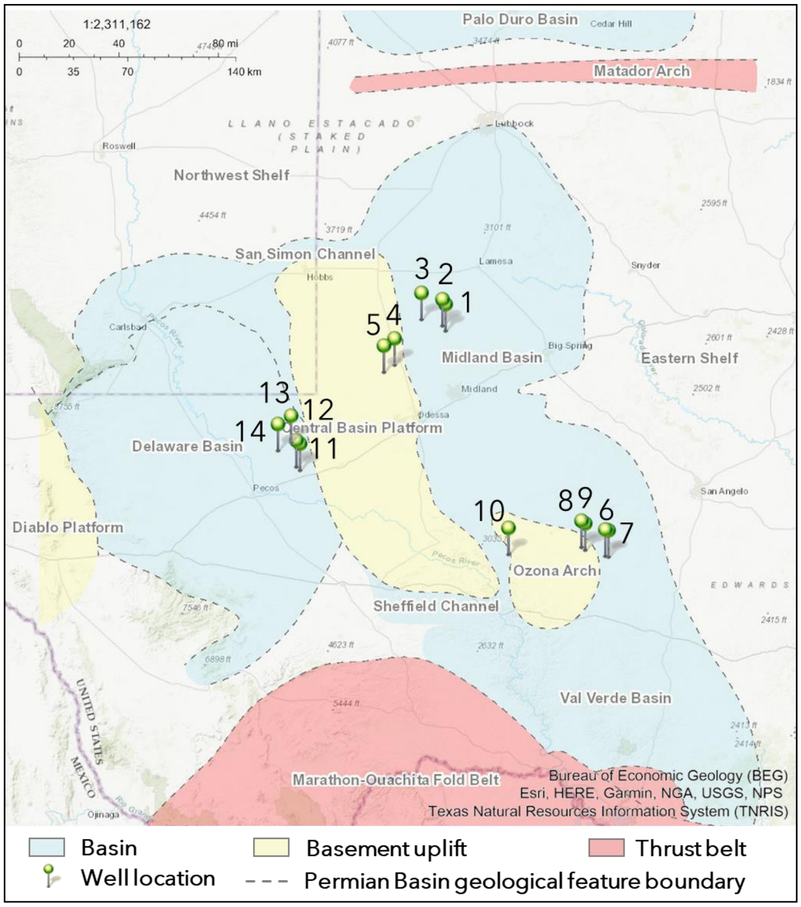

Figure S3: Permian Basin map with the distribution of oil wells, related to Table S2 and Figure 4.

Green location pins indicate the distinctive oil well locations in the different regions within the Permian Basin. Well 1-5 are located in the Northern Midland sub-basin; wells 6-10 are located in the Southern Midland sub-basin; wells 11-14 are located in the Delaware sub-basin. This map was adapted from University Lands in West Texas.

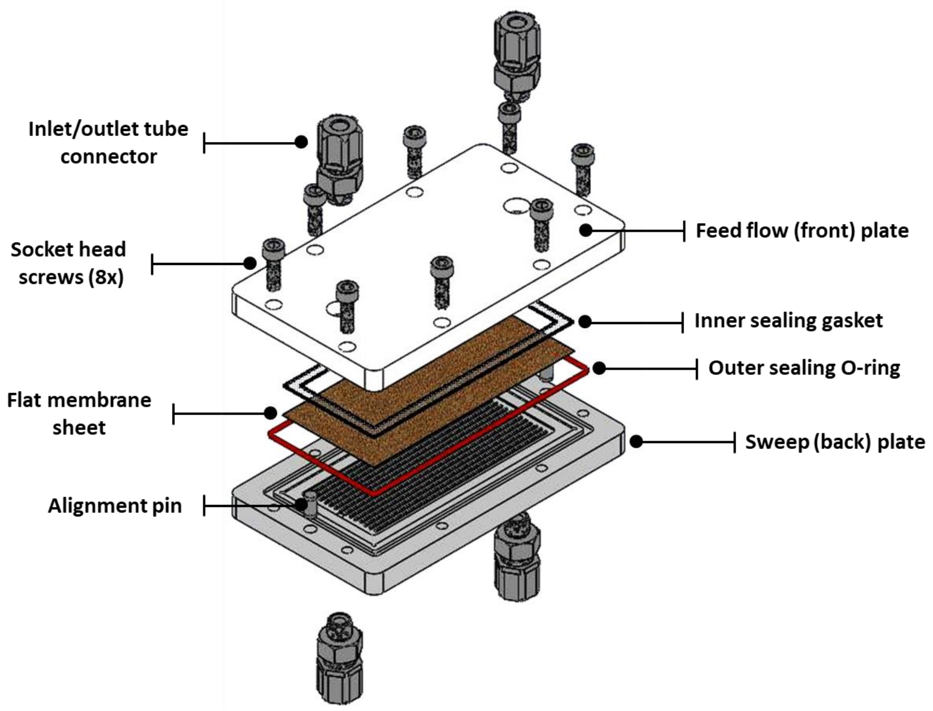

Figure S4. Schematic depiction of the filtration membrane cell utilized for deoiling PW from the Permian Basin, related to STAR Methods. Reproduced with permission from Molecule Works Inc. https://moleculeworks.com/device.

Table S2: List of wells in the Permian Basin, formations, and a representative digital photograph of the produced water, related to STAR Methods, Figure S3, and Figure S4.

CSA/glass sphere-coated membranes with a 4:1 weight ratio, loading totaling 20 mg·cm^{-2}, and thickness of 207 ± 35 µm were utilized in these measurements. The filtration membrane cell was run at 1600 L·h^{-1}·m^{-2}. These conditions were applied for the separation of PW emulsions for wells 1—14.

	Well #	Formation	Digital Photograph from Produced water from representative well in Sub-basin	Digital Photograph of Filtered water
**Northern Midland Sub-basin**	Well 1	Spraberry		
	Well 2	Spraberry		
	Well 3	Spraberry	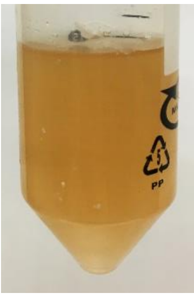	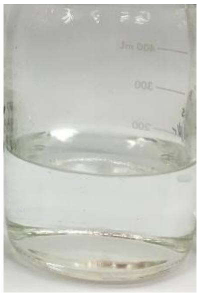
	Well 4	Wolfcamp		
	Well 5	Wolfcamp		
**Southern Midland Sub-basin**	Well 6	Wolfcamp		
	Well 7	Wolfcamp		
	Well 8	Wolfcamp	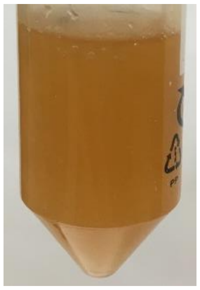	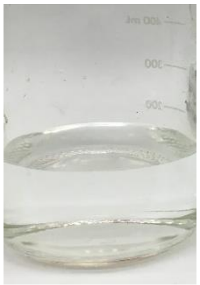
	Well 9	Wolfcamp		
	Well 10	Spraberry		
**Delaware Sub-basin**	Well 11	Spraberry		
	Well 12	Wolfcamp	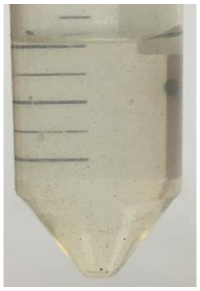	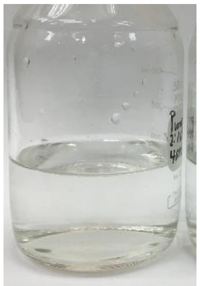
	Well 13	Wolfcamp		
	Well 14	Bone Springs		

Table S3: List of oil fractions detected in the samples from the Permian Basin with the corresponding retention times and molecular weight, related to Figure 4 and Figure S3.

Oil fraction	Retention time (min)	Molecular weight (g/mol)
C11	8.410 ± 0.010	156
C12	9.985 ± 0.005	170
C13	11.445 ± 0.005	184
C14	12.800 ± 0.000	198
C15	14.080 ± 0.000	212
C16	15.270 ± 0.010	226
C17	16.420 ± 0.000	240
C18	17.505 ± 0.005	254
C19	18.540 ± 0.010	268
C20	19.530 ± 0.010	282
C21	20.470 ± 0.010	296
C22	21.375 ± 0.015	310
C23	22.245 ± 0.015	324
C24	23.080 ± 0.010	338
C25	23.885 ± 0.015	352
C26	24.655 ± 0.015	366
C27	25.405 ± 0.015	380
C28	26.130 ± 0.010	394
C29	26.825 ± 0.015	408
C30	27.505 ± 0.015	453

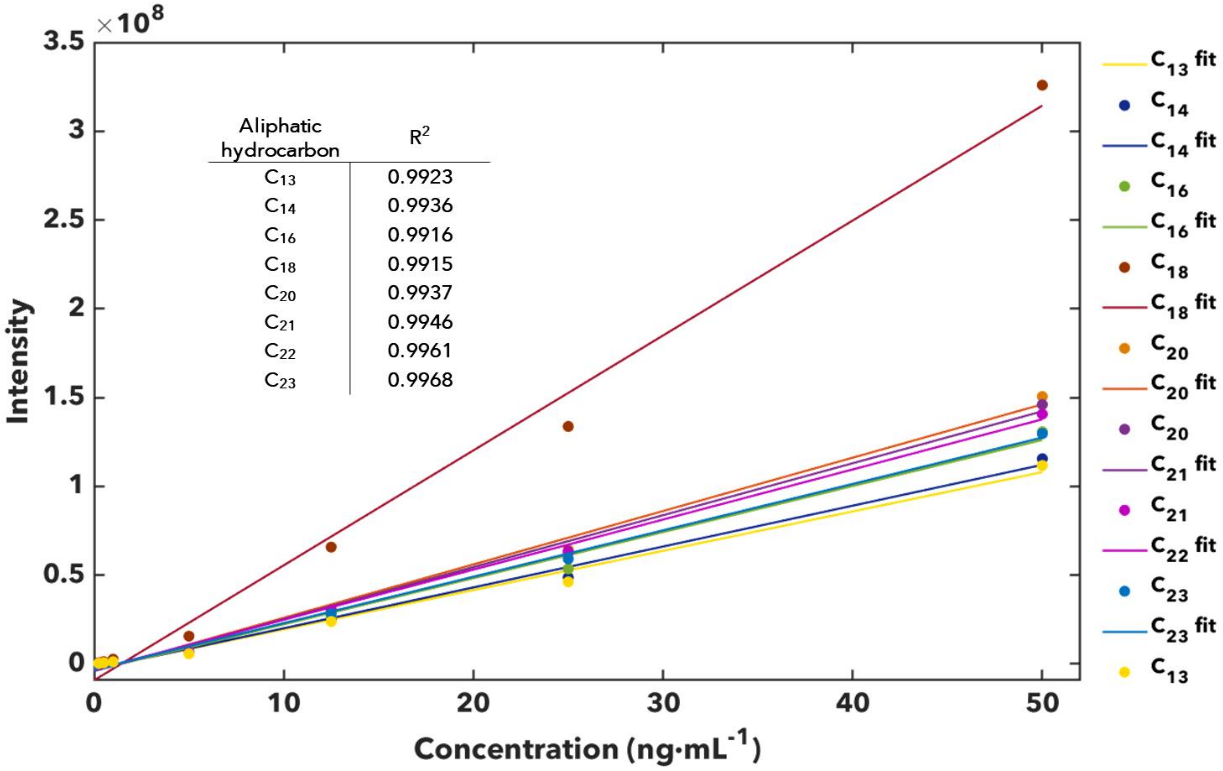

Figure S5. Calibration curves for the primary oil fractions in Well 9 ranging from C13—C23 and corresponding linear fits, related to Figure 5A and Table S4. Calibration curves were determined using a C8—C40 Alkane Calibration Standard in dichloromethane. Concentrations prepared range from 0.25—50 ng/mL.

Table S4: Concentration of oil (in ppb) remaining for the distinct oil fractions evaluated from Well 9 after separation using coated membranes with different CSA/GS formulations and total loading, related to Figure 5A and Figure S5.

*Highlighting the most abundant fractions from C13 to C23.

Total loading ($mg{\cdot}cm^{-2}$)	Aliphatic hydrocarbon	Concentration (ng/mL = pbb) (Efficiency (%))		
		Ratio of CSA/GS		
		1:1	2:1	4:1
5	C13	1.49 ± 0.25 (98.6)	1.45 ± 0.13 (98.6)	1.57 ± 0.09 (98.5)
	C14	1.61 ± 0.11 (98.7)	1.32 ± 0.03 (99.0)	1.57 ± 0.01 (98.8)
	C16	4.77 ± 0.71 (94.3)	1.45 ± 0.03 (98.3)	1.70 ± 0.03 (98.0)
	C18	6.94 ± 0.61 (83.8)	1.44 ± 0.01 (96.6)	1.68 ± 0.0004 (96.1)
	C20	18.60 ± 2.66 (54.5)	1.39 ± 0.03 (96.6)	1.67 ± 0.01 (95.9)
	C21	16.80 ± 1.72 (47.8)	ND	1.62 ± 0.003 (95.0)
	C22	18.30 ± 0.09 (41.0)	1.23 ± 0.03 (96.0)	1.44 ± 0.04 (95.3)
	C23	14.96 ± 0.58 (40.9)	ND	1.40 ± 0.02 (94.5)
10	C13	2.89 ± 0.12 (97.3)	2.59 ± 1.71 (97.6)	1.87 ± 0.12 (98.3)
	C14	3.43 ± 0.15 (97.3)	2.96 ± 2.32 (97.7)	1.66 ± 0.13 (98.7)
	C16	6.13 ± 0.22 (92.7)	4.42 ± 4.53 (94.7)	1.76 ± 0.11 (97.9)
	C18	6.48 ± 0.55 (84.9)	2.68 ± 1.78 (93.8)	1.72 ± 0.03 (96.0)
	C20	11.67 ± 0.54 (71.4)	2.97 ± 2.20 (92.7)	1.71 ± 0.08 (95.8)
	C21	9.46 ± 1.28 (70.6)	2.93 ± 2.12 (90.9)	1.65 ± 0.04 (94.9)
	C22	26.40 ± 0.40 (14.9)	2.67 ± 2.03 (91.4)	1.50 ± 0.06 (95.2)
	C23	11.60 ± 4.36 (54.2)	2.61 ± 1.97 (89.7)	1.45 ± 0.07 (94.3)
15	C13	6.21 ± 0.63 (94.2)	2.66 ± 0.34 (97.5)	1.56 ± 0.04 (98.5)
	C14	7.45 ± 0.45 (94.1)	2.87 ± 0.10 (97.7)	ND
	C16	8.09 ± 0.18 (90.3)	2.80 ± 0.15 (96.7)	1.45 ± 0.01(98.3)
	C18	7.95 ± 0.29 (81.5)	3.58 ± 0.18 (91.7)	ND
	C20	13.18 ± 0.62 (67.8)	3.96 ± 0.20 (90.3)	1.37 ± 0.02 (96.6)
	C21	12.52 ± 1.75 (61.1)	2.60 ± 0.14 (88.8)	1.40 ± 0.02 (95.6)
	C22	18.31 ± 6.76 (40.9)	3.23 ± 0.22 (89.6)	1.24 ± 0.01 (96.0)
	C23	12.03 ± 0.44 (52.5)	3.00 ± 0.16 (88.1)	ND
20	C13	1.28 ± 0.03 (98.8)	1.42 ± 0.12 (98.7)	1.34 ± 0.07 (98.7)
	C14	1.40 ± 0.13 (98.9)		
	C16	1.44 ± 0.01 (98.3)		
	C18	1.43 ± 0.004 (96.7)		
	C20	1.38 ± 0.02 (96.6)	Not detected	Not detected
	C21			
	C22	Not detected		
	C23			

Table S5: Comparison of the separation efficiency (in terms of oil concentration remaining after separation) of CSA-based membranes and CSA only substrate through the differences in oil concentrations (in ppb) for the different oil fractions in Well 9 after separation, related to Figure 5 and Figure 6.

*Highlighting the most abundant fractions from C13 to C23.

	Concentration of different aliphatic hydrocarbons in produced water and filtrate (ppb)				
	**Well 9 - Produced water**	**CSA/GS 4:1 / 20 mg·cm^{-2}**		**CSA only**	
		**After 0.5 L**	**After 1 L**	**After 0.5 L**	**After 1 L**
**C13**	75.27 ± 44.58	1.31 ± 0.02	1.32 ± 0.02	2.41 ± 0.07	1.62 ± 0.04
**C14**	85.99 ± 57.07	1.31 ± 0.01	1.34 ± 0.01	2.39 ± 0.11	1.63 ± 0.02
**C16**	60.21 ± 33.25	1.45 ± 0.01	1.47 ± 0.01	2.16 ± 0.06	1.66 ± 0.02
**C18**	27.76 ± 21.43	1.45 ± 0.01	1.45 ± 0.01	1.84 ± 0.03	1.5 ± 0.01
**C20**	30.85 ± 14.2	1.39 ± 0.03	1.39 ± 0.01	1.69 ± 0.02	1.47 ± 0.01
**C21**	26.78 ± 7.66	1.41 ± 0.03	1.4 ± 0.02	1.63 ± 0.02	1.47 ± 0.01
**C22**	24.55 ± 9.15	ND	ND	1.38 ± 0.01	1.28 ± 0.01
**C23**	21.02 ± 6.08	1.23 ± 0.01	ND	1.35 ± 0.01	1.27 ± 0.01

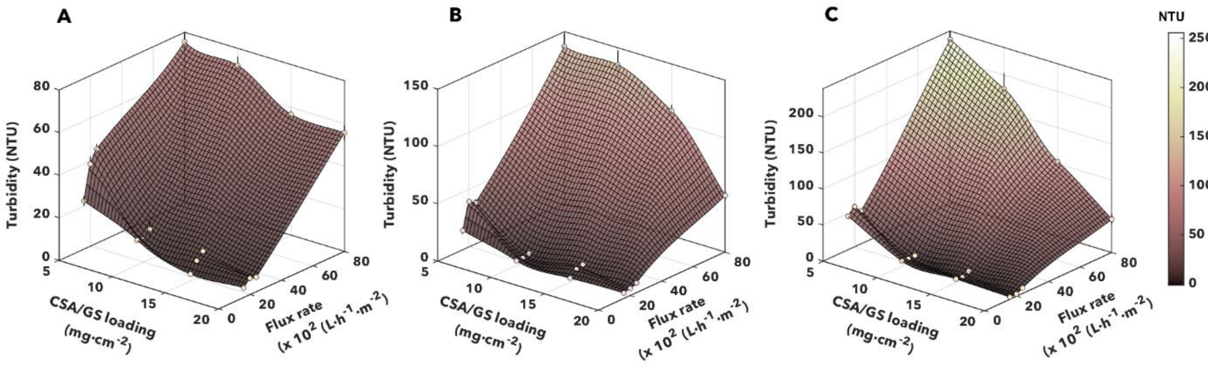

Figure S6. 3D plots for turbidity measurements measured for membranes as a function of CSA/GS loading and flux rate, related to Figure 5.
Different CSA/GS weight ratios have been evaluated: (A) 4:1 (B) 2:1 (C) 1:1.

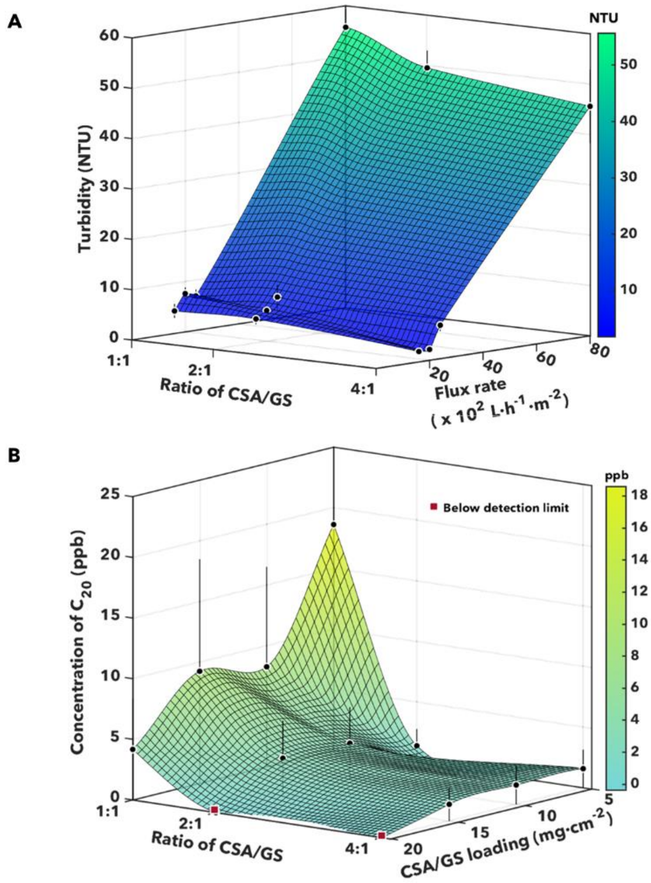

Figure S7: Optimization of functional performance for CSA-based membranes used destabilize and separate PW emulsions from the Permian Basin, related to Figure 5 and Figure S6.
(A) 3D plot of turbidity of filtered water as a function of the ratio of CSA/GS and flux rate (1600—8000 L·h^{-1}·m^{-2}) (at a CSA/GS loading of 20 mg·cm^{-2} and thickness of 207 ± 35 µm) (B) 3D plot of oil concentration (in ppb) for filtered water as a function of the ratio of CSA/GS and CSA/GS loading (in mg·cm^{-2}). The concentration of oil highlighted is from C20, one of the most dominant fractions throughout the water samples.

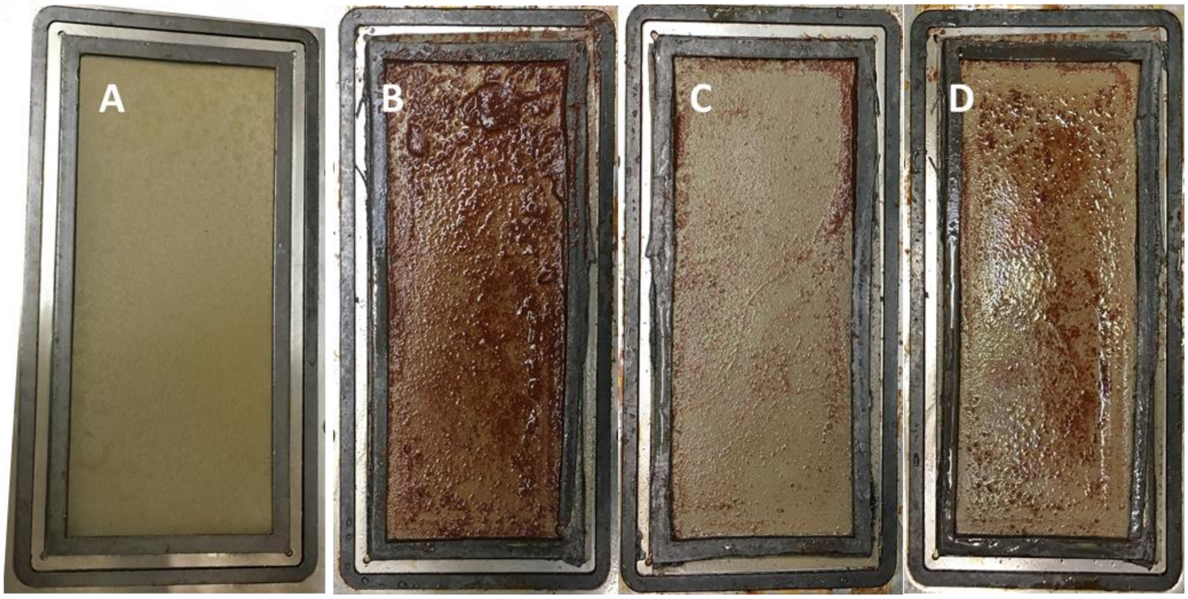

Figure S8. Digital photographs of CSA/GS membranes, related to Figure 6. A) pristine membrane hydrated for 24 h; B) membrane after filtration of 500 mL of PW; C) membrane after backwash with 5 mL water after first separation cycle; and D) membrane after backwash with 5 mL water after 10th separation cycle.

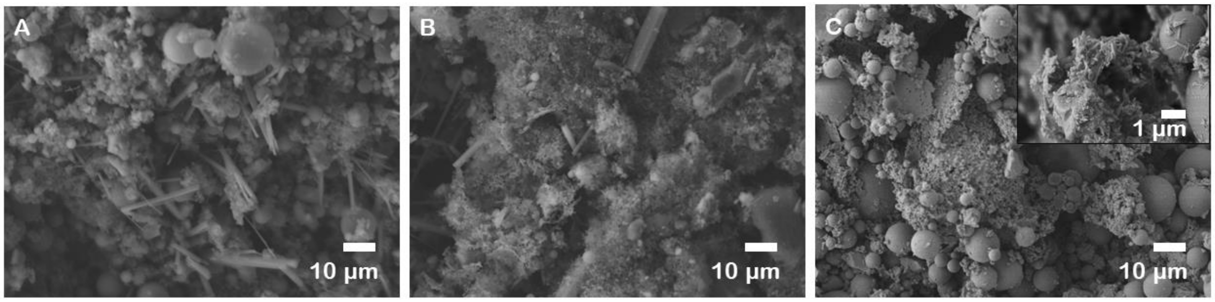

Figure S9. Scanning electron micrographs of 4:1 CSA/GS membranes depicting the changes in morphology and ettringite presence after separation and backwash, related to Figure 6 and Figure S8. A) pristine membrane with 24 h hydration, B) after initial separation of PW stream, and C) after backwashing with 5 mL water.

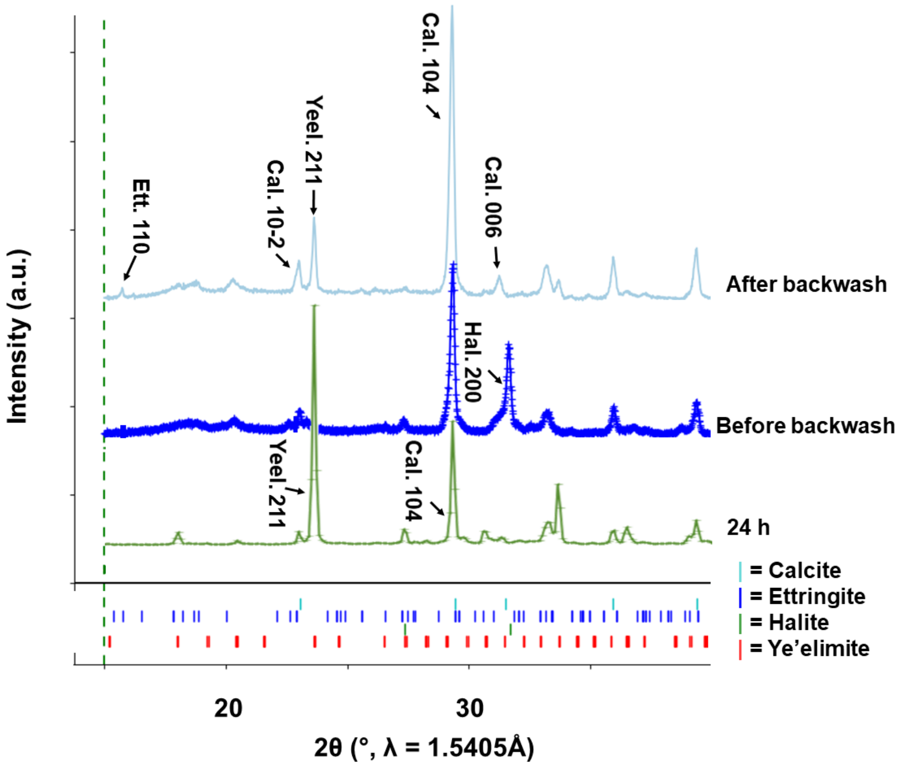

Figure S10. Powder XRD patterns for hydrated CSA (24 h), before backwash and after backwash with 10 mL water, related to Figure 6 and Figure S9.

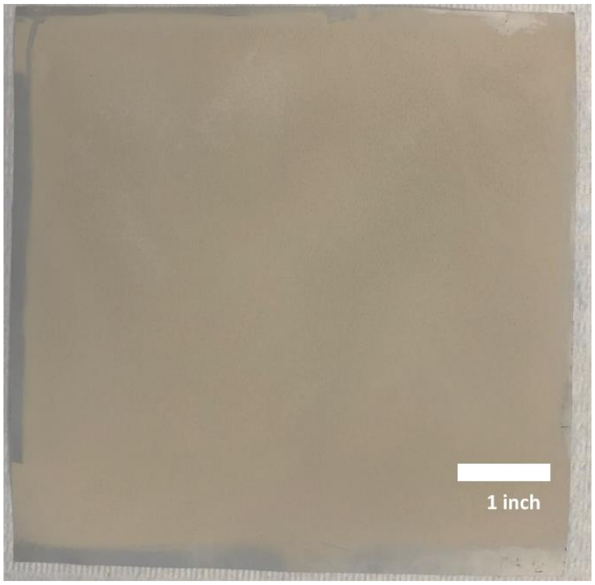

Figure S11. Digital photographs of as prepared 4:1 CSA/GS membrane, related to STAR Methods. A 6''×6'' membrane as an exemplar of scalable manufacturing.
